# Digital and Interactive Health Interventions Minimize the Physical and Psychological Impact of Breast Cancer, Increasing Women’s Quality of Life: A Systematic Review and Meta-Analysis

**DOI:** 10.3390/cancers14174133

**Published:** 2022-08-26

**Authors:** Esteban Obrero-Gaitán, Irene Cortés-Pérez, Tania Calet-Fernández, Héctor García-López, María del Carmen López Ruiz, María Catalina Osuna-Pérez

**Affiliations:** 1 Department of Health Sciences, University of Jaén, Campus Las Lagunillas s/n, 23071 Jaén, Spain; 2 Clínica Vitaudio Linares, Baños Street 16, 23700 Linares, Spain; 3 Physical Therapy and Medicine, Department of Nursing, University of Almeria, Road Sacramento s/n, 04120 Almeria, Spain

**Keywords:** breast cancer, women, virtual reality, smartphone apps, pain, anxiety, depression, quality of life, upper extremity, meta-analysis

## Abstract

**Simple Summary:**

The development of digital and virtual devices for therapeutic purposes has been widely studied in recent years. There is a growing scientific interest in analyzing the effects of digital and interactive health interventions (DIHIs) in the management of different neurological and musculoskeletal pathologies, as well as breast cancer (BC). DIHIs include the use of a wide variety of virtual reality devices, as well as smartphone apps and games, to reduce the impact of BC signs and symptoms on women. In the present review, we assessed the effect of DIHIs, in comparison to conventional interventions or usual care, on improving the motor control and function of the upper extremities affected by lymphedema after BC surgery, including pain, anxiety, depression, and different dimensions of quality of life. In addition, we investigated which type of DIHI was more useful for women with BC: virtual reality devices or smartphone apps.

**Abstract:**

Digital and interactive health interventions (DIHIs), such as virtual-reality-based therapy (VRBT) and smartphone-app-based therapy (SABT), may be useful for reducing the impact of the signs and symptoms of breast cancer (BC) in women. The aim of this meta-analysis was to explore the effect of DIHIs on improving pain, anxiety, depression, quality of life (QoL), and upper extremity (UE) disability-related lymphedema in women with BC. Methods: We searched PubMed Medline, Web of Science, Scopus, CINAHL, Physiotherapy Evidence Database, and SciELO for the period ending February 2022. We included studies that assessed the effect of DIHIs on UE motor disability, pain, anxiety, depression, and QoL in women with BC. The effect size was calculated using Cohen’s standardized mean difference (SMD) and its 95% confidence interval (95% CI). Results: Twenty studies providing data from 1613 women with BC were included. With respect to UE disability, DIHIs increased flexion (SMD, 1.92; 95%CI: −1.16, 2.68), abduction (SMD, 1.66; 95%CI: 0.91, 2.42), external rotation shoulder range of motion (SMD, 1.1; 95%CI: 0.36, 1.85), UE function (SMD, −0.72; 95%CI: −1.31, −0.13), and handgrip strength (SMD, 0.4; 95%CI: 0.21, 0.59). DIHIs reduced pain (SMD, −0.8; 95%CI: −1.31, −0.26), anxiety (SMD, −1.02; 95%CI: −1.71, −0.34), and depression (SMD, −1.57; 95%CI: −3.1, −0.08). Finally, DIHIs increased overall health (SMD, 0.6; 95%CI: 0.31, 0.89). Conclusions: Right at the end of therapy, DIHIs are effective at improving UE function, pain, anxiety, depression, and QoL in women with BC. VRBT has a greater effect than SABT for the assessed outcomes.

## 1. Introduction

Breast cancer (BC) is the most common cancer in the majority of countries, representing 11.7% of all cancer cases, and it is the fifth-leading cause of cancer mortality worldwide (mortality rate of 6.9%) [1]. BC constitutes the second most common cause of mortality (685,000 deaths in 2020), morbidity, and associated disability in women [2]. In 2020, 2.3 million new cases of BC were diagnosed worldwide [3], and this incidence may increase to around 4.4 million new cases by 2070 [4]. However, advances in the diagnosis, prevention, and treatment of BC have increased the survival rate in recent years [5]; currently, the average 5-year survival rate is greater than 85% [6].

Women diagnosed with BC experience physical and psychosocial adverse effects during treatment that can reduce the effect of their received therapies and worsen their prognosis and their quality of life [7]. Long-term cancer survivors show a significant reduction in quality of life even 2 years after diagnosis [8,9]. Pain during disease or therapy is the most disabling symptom for women with BC (32–47%) [10]. Sometimes, pain is reported 3 years after the end of the therapy, having become neuropathic and chronic pain that may cause sensorimotor disturbances in the body, especially in the upper extremities. Recent studies have shown both central sensitization patterns that produce changes in pain sensitivity stimulus [11] and rotator cuff damage in the shoulder and neck area in women who survived BC after post-mastectomy surgery [12]. BC-related lymphedema is a sensorimotor disabling sign that is accompanied by pain, upper extremity motor disorders [13,14], and skin alterations [15]; it appears in 14–54% of survivors (almost one in five BC survivors) after axillary surgery or irradiation [16,17].

Women with BC can experience emotional distress and have subsequent psychological needs [18] related to the duration of the disease, surgical or chemical therapies, and impaired body image [19]. Anxiety and depression are two representative emotional disorders in these patients [20]. One in three women with BC presents with an anxiety disorder more than 5 years after diagnosis [21,22], which may negatively affect treatment, recurrence, and mortality [23]. A negative psychological status impairs social relationships, intimacy in couples, and work life. Recent studies have associated this psychological impact with lower levels of white blood cell activity and antibodies and an increased stress hormone response [24]. Therefore, it is crucial to develop therapies that reduce negative feelings related to the fear of disease progression and the risk of recidivism and that promote positive emotions and better coping.

The latest technological advances have allowed the development of digital and interactive health interventions (DIHIs) for women with BC. The use of virtual reality (VR) devices and smartphone apps has allowed the development of videogame-based interventions that reduce the impact of different pathologies [25,26], including BC [27], through the practice of various physical and cognitive exercises. On the one hand, virtual-reality-based therapy (VRBT) allows patients to interact with and immerse themselves in a computer-generated environment that can make them feel as if they are actually present in that world, offering immersive, semi-immersive, and non-immersive experiences depending on the type of VR. This modality provides engaging multi-sensory entertainment and real-time feedback [28,29]. On the other hand, the use of smartphone apps or game-based therapy allows the development of educational apps and mobile videogames that support the practice of supervised physical and mental exercises using accessible and active virtual environments [30]. Recent studies suggest that VRBT [31] and smartphone-app-based therapy (SABT) [32] have high rates of acceptance among women with BC and may be useful for increasing quality of life and improving self-management. These tools may use videogames developed for commercial entertainment or games specifically designed for health interventions. The game-playing experience is very absorbing, and virtual reality devices and smartphones enhance adherence to the therapy and reduce some of the negative aspects of the disease and its treatments [33].

To date, previous reviews have assessed the effect of DIHIs (VRBT [34,35,36] and SABT [32,37]) with promising results. However, the large heterogeneity in the design of the studies included and the fact that studies with only one group were combined with those with two or more groups may have minimized the impact of the results. Reviews that assessed the use of SABT included apps that monitor symptoms, and it is necessary to assess the effectiveness of apps that include exercises using games or supervised videos. However, currently, there is no synthesized evidence that groups VRBT and SABT together in order to assess their effect on women with BC, and no reviews have assessed the impact of DIHIs on different dimensions of quality of life. The objective of this study was to analyze the effect of DIHIs on pain, anxiety, depression, quality of life, and upper extremity disability in women with BC. Secondly, we wanted to investigate which type of digital and interactive health intervention (VRBT or SABT) might be more effective for the recovery of each disabling symptom.

## 2. Materials and Methods

We registered this review in PROSPERO (CRD42022301708). The authors followed the guidelines of the Preferred Reporting Items for Systematic Reviews and Meta-Analyses (PRISMA) statement [38].

### 2.1. Literature Search

To perform this review, we searched PubMed Medline, Scopus, CINAHL Complete, Web of Science (WOS), Physiotherapy Evidence Database (PEDro), and SciELO. To identify all references for potential inclusion, we screened reference lists from published studies, in congress abstracts, and conference proceedings for the period ending February 2022. To build the chain literature search used in each database, we selected three conditions for the PICOS system [39]. For the population (women diagnosed with BC) we used “breast cancer” and “breast neoplasm”; for the experimental intervention therapy (digital and interactive health interventions) we used “virtual reality”, “mobile applications”, and “videogames”; and for the study design, we selected studies with an experimental design with two groups. Medical Subjects Headings (MeSH) nomenclature was used to select the main keywords employed in the search and entry terms. The conditions were joined with the Boolean operator “AND”, while the keywords for each condition used “OR”. This search strategy was not restricted by publication date or language. Two authors independently designed and carried out a literature search, and a third author supervised this phase. The search strategies used for each database are shown in Table 1.

### 2.2. Study Selection—Inclusion and Exclusion Criteria

Two authors independently performed an initial selection of studies according to the titles and abstracts of all identified studies. A study was examined in detail if at least one of the authors selected it by title or abstract. Finally, a third author was consulted as to whether to include in the review questionable preselected studies.

A study was included in this meta-analysis if it met all these inclusion criteria: study design (randomized and non-randomized experimental studies); population (women diagnosed with BC); experimental intervention group (VRBT or SABT); control intervention group (conventional therapy or usual care); and outcomes (see Section 2.5). In addition, all studies selected provided quantitative data regarding the outcomes of interest to perform the quantitative synthesis. The exclusion criteria proposed were: studies in which the sample consisted of patients with different cancers (not only BC) and studies in which all groups received digital and interactive health interventions.

### 2.3. Data Extraction

Data were independently compiled by two authors using an Excel data collection form, and disagreements were referred to a third author. We extracted data related to overall characteristics (authorship, publication date and country, type of study, and funding information), characteristics related to patients (total number of participants and groups, age, gender, and time since diagnosis), characteristics related to interventions (type of device used in the experimental intervention, type of control intervention, and dose of the interventions), and data on the outcomes (quantitative data from the assessment of each outcome at the conclusion of the therapy). When possible, these data were the mean and its standard deviation; when these were not available, we extracted the interquartile range, range, and standard error, and we used standardized transformations to estimate the standard deviation [39,40].

### 2.4. Outcomes

The outcomes analyzed in this meta-analysis were related to disabling symptoms present in women with BC. These outcomes were UE disability-related lymphedema (shoulder range of motion and shoulder muscle strength, lymphedema symptoms, handgrip strength, and UE function), pain, anxiety, depression, and different dimensions of quality of life (overall, physical, mental, emotional, social functioning, and vitality).

### 2.5. Analysis of the Risk of Bias of the Studies Included and the Quality of the Evidence of the Findings

The risk of bias was assessed using the Cochrane Collaboration Risk of Bias Tool [39]. This tool assesses six biases (selection, performance, detection, attrition, reporting, and other bias), classifying the risk of bias as low, high, or uncertain (information not contained in studies) [41].

The quality of evidence was assessed using the Grading of Recommendations Assessment, Development, and Evaluation (GRADE) statement [42] and Meader’s checklist [43]. The risks of bias, imprecision, indirectness, inconsistency, and publication bias were taken into account to determine the quality of evidence: (1) high, if the findings were robust and generalizable; (2) moderate, when new research could modify our findings; (3) low, if the level of confidence in our findings was vague; and (4) very low, when our findings were uncertain. Two authors participated in these evaluations independently, and another author resolved disagreements.

### 2.6. Statistical Analysis

Comprehensive Meta-Analysis version 3.0 (Biostat, Englewood, NJ, USA) [44] was used to carry out the quantitative synthesis. A meta-analysis was only performed when more than one study reported data for that analysis. We used a fixed or random effect model according to the level of heterogeneity found, following the guidelines of Dersimonian and Laird [45]. The pooled effect was calculated using Cohen’s standardized mean difference (SMD) [46] and its 95% confidence interval (95% CI). Effect size was classified as no effect (SMD, 0), small (SMD, 0.2), medium (SMD, 0.5), or large (SMD > 0.8) [47]. We calculated the mean difference (MD) between groups to compare our results to the minimal clinically important difference (MCID) value for this measurement tool. MCID is defined as the “smallest difference in score in the domain of interest which participants perceive as beneficial and which would mandate, in the absence of troublesome side effects and costs, a change in the patient’s management” [48]. Our findings were displayed in forest plots [49]. The risk of publication bias was assessed with funnel plots, which can be symmetric (no risk) or asymmetric (risk), with the *p*-value for the Egger test [50] and with trim-and-fill estimation [51]. According to Rothman, when variation between the original and estimated effects with trim-and-fill is higher than 10%, the level of evidence is downgraded one level [52]. When calculating the degree of inconsistency (I^2^), heterogeneity can be low (<25%), moderate (25–50%), or large (>50%). In addition, heterogeneity is confirmed if *p* for the Q-test < 0.01 [53,54].

### 2.7. Sensitivity and Subgroup Analyses

A one-study-removed approach was used to perform the sensitivity analysis. Meta-regression was performed to assess the effect according to the different study designs included: randomized controlled trials and quasi-experimental studies [39]. In addition, we performed subgroup analyses to assess the effect of different DIHIs on each outcome (VRBT vs. controls and SABT vs. controls).

## 3. Results

### 3.1. Study Selection Process

**Figure 1:**
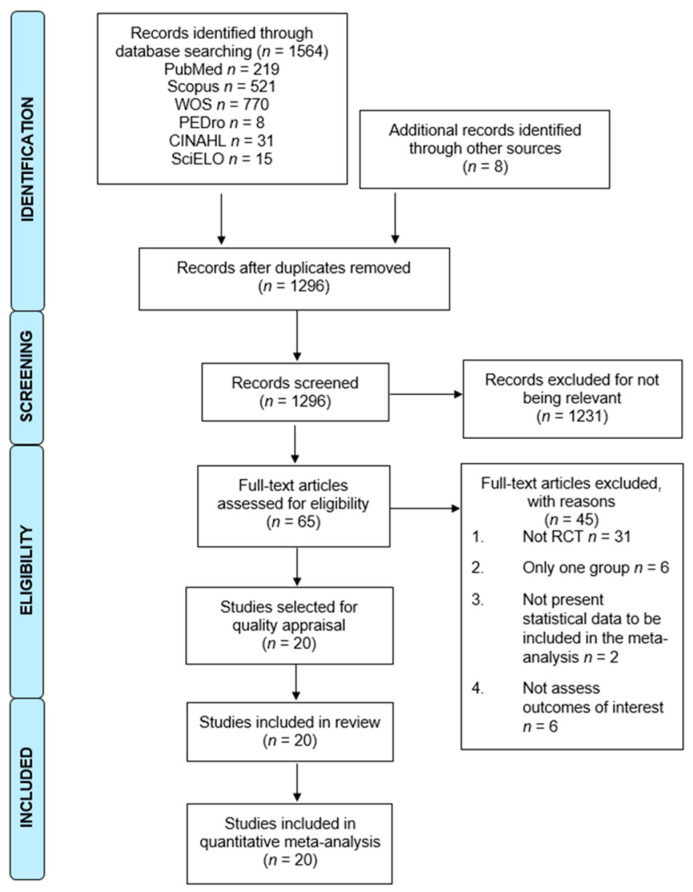
PRISMA flow chart of the study selection process.

The PRISMA flow chart (Figure 1) displays the results of the study selection process. A total of 1564 studies were retrieved from the databases, and a further 8 studies were retrieved from complementary sources. After duplications were removed (*n* = 276), 1296 studies were screened by title/abstract. Of those, 1231 studies were excluded by title/abstract and 45 for not meeting the inclusion criteria (reasons are given in Figure 1). Finally, 20 studies [55,56,57,58,59,60,61,62,63,64,65,66,67,68,69,70,71,72,73,74] were included in this review.

### 3.2. Main Characteristics of the Studies Included

Twenty studies included in our systematic review with meta-analysis [55,56,57,58,59,60,61,62,63,64,65,66,67,68,69,70,71,72,73,74] provided 92 independent comparisons. These studies were carried out between 2015 and 2022 in countries such as China [59,60,66,72], Korea [61,63,65], Japan [57,62], Turkey [69,71,73], Egypt [56,68], Jordan [67], Iran [74], Italy [70], Australia [58], the United States [64], and Mexico [55]. Of the studies included, 14 were randomized controlled trials [57,59,60,61,62,64,66,67,68,69,71,72,73,74] and 6 were quasi-experimental pre–post studies [55,56,58,60,63,65], all with one comparison group. The included studies provided data from 1613 patients with BC (mean age of 50.68 ± 3.7 years old and all female). The mean number of participants per study was 81. A total of 783 subjects (50.12 ± 3.64 years old) were part of an experimental intervention group and 830 (51.24 ± 3.8 years old) were in a control or comparison intervention group. Patients in the experimental intervention groups received DIHIs using immersive and non-immersive VRBT [55,56,58,59,60,66,67,68,73] or SABT [57,61,62,63,64,65,69,71,72,74]. Patients in the control intervention groups underwent conventional therapies [56,59,60,66,68,70,73] or care [55,57,58,61,62,63,64,65,67,69,70,71,72,74]. DIHIs were applied for a time period ranging between 1 day and 12 weeks. The number of sessions per week was between one and five, and the duration of DIHI exposition was between 15 and 60 min. All studies provided data that allowed the assessment, at the conclusion of therapy, of one or more of the variables of interest: upper extremity disability-related lymphedema, pain, anxiety, depression, or quality of life. Finally, as complementary information, 12 studies received external funding to perform the research [58,61,62,63,64,65,67,69,70,71,72,74]. Table 2 shows the detailed characteristics of the studies included.

### 3.3. Risk of Bias of the Studies Included

**Figure 2:**
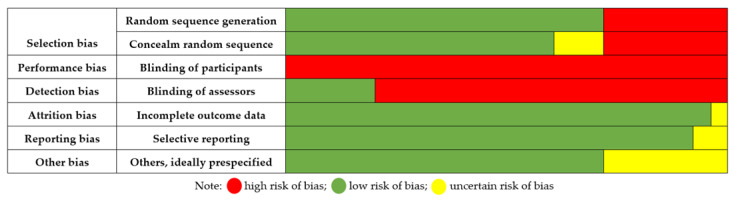
Cochrane risk of bias tool assessment.

Table 3 and Figure 2 show the assessment of the risk of bias using the Cochrane ROB tool. The two types of risk of bias found to be present were performance and detection bias. The participants of all the included studies were not blinded due to the characteristics of the therapies, and in only 20% of the studies were the assessors blinded. The risk of selection bias was low due to 70% of articles being randomized controlled trials, and no risk was found in random sequence generation or concealment sequence generation. Finally, due to the nature of quasi-experimental studies, other biases could be present due to comparability between groups.

### 3.4. Outcome Measurements

First, we assessed four outcomes related to UE disability in women with BC and lymphedema. Shoulder range of motion was assessed with goniometry in degrees; shoulder muscle strength in kilograms; lymphedema symptoms from limb volume measurements in milliliters; handgrip strength with a dynamometer, and upper extremity disability with data from the Disability of the Arm, Shoulder, and Hand (DASH) questionnaire and the Quick DASH-9 Scale. Pain was assessed with data from the Visual Analog Scale (VAS) and the SF-36 body pain dimension. Anxiety was assessed with data from the following: the State-Trait Anxiety Inventory (STAI), the Hospital Anxiety and Depression Scale (HADS)-anxiety dimension, the State Anxiety Inventory (SAI), and the NCCN Distress Thermometer Scale. Depression was assessed with data from the Profile of Mood State-short version, the HADS-depression dimension, and the Beck Depression Inventory (BDI). Finally, quality of life was assessed with data from the FACTES quality of life scale, the SF-36, the World Health Organization Quality of Life-BREF Scale, the EuroQoL-5D, and the European Organization for Research and Treatment of Cancer Quality of Life Questionnaire Core 30 (EORTC QLQ-C30).

### 3.5. Quantitative Synthesis

Our systematic review integrated meta-analyses to assess the effect of DIHIs on outcomes related to upper-extremity-related lymphedema, pain, anxiety, depression, and quality of life. Table 4 summarizes the main findings of these meta-analyses.

#### 3.5.1. Shoulder Range of Motion (Flexion, Abduction, and External Rotation)

**Figure 3:**
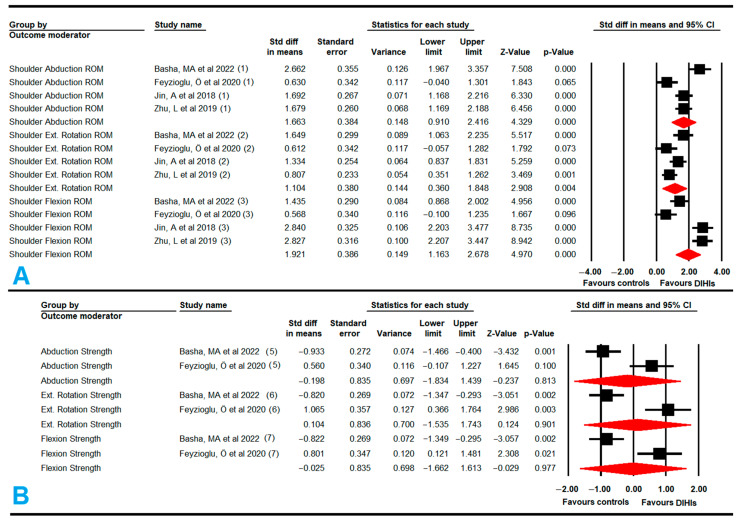
Forest plot of the effect of DIHIs on shoulder range of motion (**A**) and shoulder strength movements (**B**) [60,66,68,73].

Four studies [60,66,68,73] with four independent comparisons provided data from 248 participants (62 per study) to assess the effect of non-immersive VRBT compared to conventional physical training. Low-quality evidence of a large effect on flexion (SMD, 1.92; 95% CI: −1.16, 2.68; *p* < 0.001), abduction (SMD, 1.66; 95% CI: 0.91, 2.42; *p* < 0.001), and external rotation shoulder range of motion (SMD, 1.1; 95% CI: 0.36, 1.85; *p* = 0.004) favored non-immersive VRBT (Figure 3, Table 4). A risk of publication bias and inconsistency was detected in the abduction range of motion meta-analysis due to an asymmetric funnel plot and a 19% variation after trim-and-fill estimation (adjusted SMD, 1.35) (Supplementary Figures S1–S3 show funnel plots of the three meta-analyses). No heterogeneity was shown in the meta-analyses. The sensitivity analysis did not report substantial variations with respect to the original SMD.

#### 3.5.2. Shoulder Muscle Strength (Flexion, Abduction, and External Rotation)

Two studies [68,73] with two independent comparisons assessed the effect of non-immersive VRBT in comparison to conventional physical training with data from 86 subjects (43 per study). No statistically significant differences were found between non-immersive VRBT and conventional physical training to improve the strength in flexion (SMD, −0.03; 95% CI: −1.66, 1.66; *p* = 0.97), abduction (SMD, −0.2; 95% CI: −1.83, 1.44; *p* = 0.81), or external rotation shoulder movements (SMD, 0.1; 95% CI: −1.54, 1.74; *p* = 0.9) (Figure 3, Table 4). The risk of publication bias was not assessed due to software characteristics, so it is important to consider the possibility that such a bias may exist. Heterogeneity was not present in any meta-analysis. The sensitivity analysis did not show variation with respect to the original effect.

#### 3.5.3. Excess Limb Volume in the Affected Upper Extremity with Lymphedema

**Figure 4:**
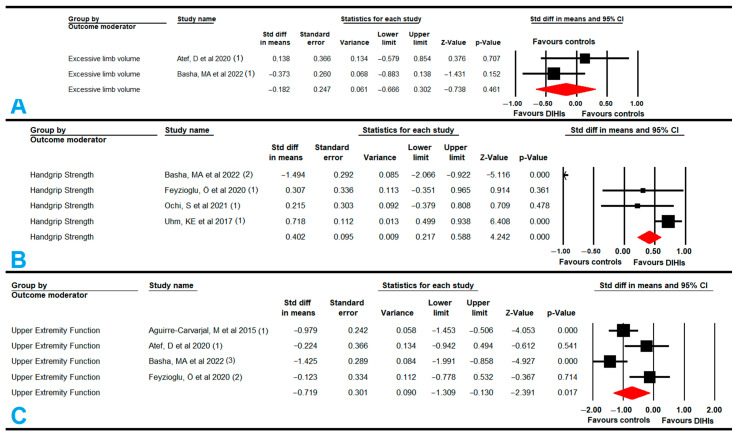
Forest plot of the effect of digital and interactive health interventions on excessive limb volume (**A**), handgrip strength (**B**), and upper extremity function (**C**) [55,56,62,65,68,73].

Two studies [56,68] with two independent comparisons provided data from 90 participants (45 per study) to assess the effect of non-immersive VRBT on reducing the excess limb volume in survivors with lymphedema, in comparison to conventional physical training. No statistically significant differences were found between therapies (SMD, −0.18; 95% CI: −0.66, 0.3; *p* = 0.46) (Figure 4, Table 4). Heterogeneity was not present, and the risk of publication bias was taken into account, although it could not be estimated. The sensitivity analysis did not show differences according to study design; studies were either randomized controlled trials (SMD, 0.14; 95% CI: −0.58, 0.84; *p* = 0.7) or quasi-experimental (SMD, −0.37; 95% CI: −0.88, 0.14; *p* = 0.15).

#### 3.5.4. Handgrip Strength

Four studies [62,65,68,73] with four independent comparisons provided data from 477 participants (119.25 per study) to assess the effect of DIHIs (SABT and VRBT videogames) on increasing handgrip strength. Very-low-quality evidence of a medium effect (SMD, 0.4; 95% CI: 0.21, 0.59; *p* < 0.001) favored DIHIs compared to conventional therapy (Figure 4, Table 4). The risk of publication bias was high (asymmetric funnel plot; Egger *p* = 0.15; 65% variation after trim-and-fill estimation (adjusted SMD, 0.67)) (Supplementary Figure S4). The heterogeneity level was high (I^2^ = 67%; *p* = 0.001). The sensitivity analysis evidenced changes in effect size when Uhm’s study was excluded due to a large sample size with respect to the other studies [65].

Subgroup analyses showed that the practice of physical exercise using SABT [62,65] increased handgrip strength more than conventional therapy (SMD, 0.66; 95% CI: 0.45; 0.87; *p* < 0.001). In contrast, exercises integrated into conventional therapy training using proprioceptive neuromuscular facilitation or resistance exercises [68,73] were more effective than non-immersive VRBT (SMD, −0.72; 95% CI: –1.15, –0.28; *p* = 0.001) for improving handgrip strength.

#### 3.5.5. Function and Disability of the Affected Upper Extremity with Lymphedema

Four studies [55,56,68,73] with four independent comparisons provided data from 203 participants (51 per study) to assess the effect of non-immersive VRBT, with respect to conventional therapy or care, on reducing disability in UE function. Low-quality evidence of a medium-large effect (SMD, −0.72; 95% CI: −1.31, −0.13; *p* = 0.017) favored non-immersive VRBT for decreasing disability in UE function (Figure 4, Table 4). No risk of publication bias and no heterogeneity were present in the meta-analysis (Supplementary Figure S5). The sensitivity analysis did not find statistically significant differences according to study design; the two types of studies analyzed were randomized controlled trials (SMD, −0.78; 95% CI: −2.1, 0.5; *p* = 0.23) and quasi-experimental designs (SMD, −0.65; 95% CI: −1.39, 0.08; *p* = 0.08).

#### 3.5.6. Pain

**Figure 5:**
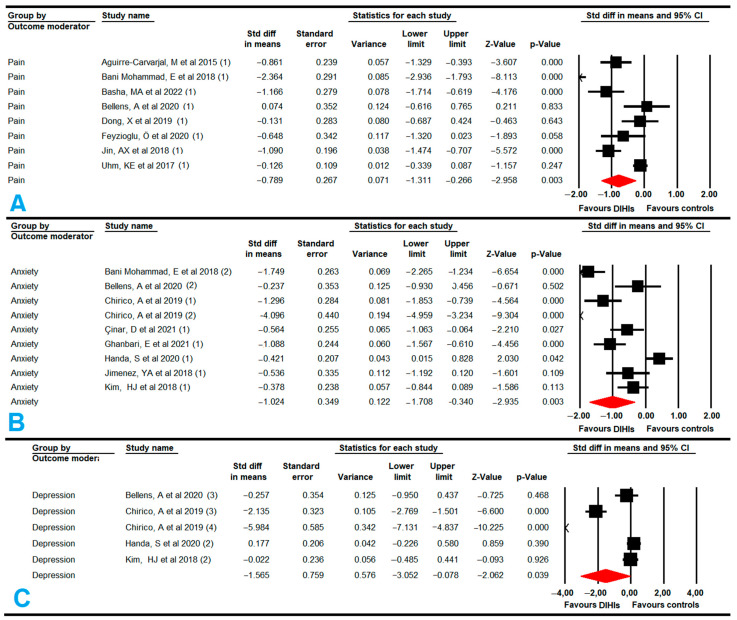
Forest plot of the effect of digital and interactive health interventions on pain (**A**), anxiety (**B**), and depression (**C**) [55,57,58,59,61,65,67,68,69,70,72,73,74].

Eight studies [55,59,65,67,68,69,72,73] with eight independent comparisons provided data from 758 participants (95 per study) to assess the effect of DIHIs with SABT and VRBT on reducing pain in women with BC. Our findings showed moderate-quality evidence of a large effect of DIHIs (SMD, −0.8; 95% CI: −1.31, −0.26; *p* = 0.003) reducing pain in comparison to conventional training or care (Figure 5, Table 4). Our findings presented a risk of publication bias (asymmetric funnel plot; Egger *p* = 0.09; 17% variation after trim-and-fill estimation (adjusted SMD, 0.9)), but no heterogeneity (Supplementary Figure S6). The sensitivity analysis showed no statistically significant differences according to study design; studies were either randomized controlled trials (SMD, −0.9; 95% CI: −1.55, −0.25); *p* = 0.007) or quasi-experimental (SMD, −0.46; 95% CI: −1.18, 0.26); *p* = 0.21). Additionally, we estimated that DIHIs could achieve a reduction of three points on the VAS for pain (MD = −3.26; 95% CI: −5.55, −1; *p* = 0.005).

Subgroup analyses found that non-immersive and immersive VRBT [55,59,67,68,69,73] produced a large effect in terms of reducing pain (SMD, −1.03; 95% CI: −1.52, −0.54; *p* < 0.001) in comparison with conventional therapy or care. Pain was able to be reduced by 3.83 points (95% CI: −5.88, −0.9; *p* = 0.008) when VRBT was used. However, no statistically significant differences were found between SABT and conventional training (SMD, −0.13; 95% CI: −0.94, 0.68; *p* = 0.756), although this finding must be considered with caution due to the low number of studies that provided data [65,72].

#### 3.5.7. Anxiety

Eight studies [57,58,61,67,69,70,71,74] with eight independent comparisons provided data from 660 participants (73 per study) to assess the effect of DIHIs on reducing anxiety. Low-quality evidence of a large effect (SMD, −1.02; 95% CI: −1.71, −0.34; *p* = 0.003) favored DIHIs in comparison to conventional therapy (Figure 5, Table 4). The risk of publication bias (asymmetric funnel plot; Egger *p* = 0.02; 32% variation after trim-and-fill estimation (adjusted SMD, −1.35)) (Supplementary Figure S7) and heterogeneity were moderate (I^2^ = 32%; *p* = 0.16) in these findings. The sensitivity analysis did not report statistically significant differences when quasi-experimental designs were excluded (SMD, −1.09; 95% CI: −1.84, −0.33; *p* = 0.005).

Subgroup analyses found that VRBT [67,69,70] produced a large effect in terms of reducing anxiety (SMD, −1.79; 95% CI: −2.7, −0.91; *p* < 0.001) in comparison with conventional therapy or care. In addition, significant differences were found between SABT [57,58,61,71,74] (SMD, −0.42; 95% CI: −1.12, −0.01; *p* = 0.047) and conventional training.

#### 3.5.8. Depression

Four studies [57,61,69,70] with five independent comparisons provided data from 402 subjects (84 per study) to assess the effect of DIHIs on reducing depressive symptoms compared to conventional training or care. Very-low-quality evidence of a large effect (SMD, −1.57; 95% CI: −3.1, −0.08; *p* = 0.039) favored DIHIs (Figure 5, Table 4). The risk of publication bias was high (asymmetric funnel plot; Egger *p* = 0.01; 29% variation after trim-and-fill estimation (adjusted SMD, −2.05)) (Supplementary Figure S8) with moderate heterogeneity (I^2^ = 46%; *p* = 0.06). The sensitivity analysis did not show variation with respect to the original effect.

Subgroup analysis showed statistically significant differences favoring VRBT [69,70] vs. conventional training or care (SMD = −2.7; 95% CI: −4.39, −0.99; *p* = 0.002), but not between SABT [57,61] and conventional training or care (SMD, 0.08; 95% CI: −1.94, 2.1; *p* = 0.94).

#### 3.5.9. Quality of Life

**Figure 6:**
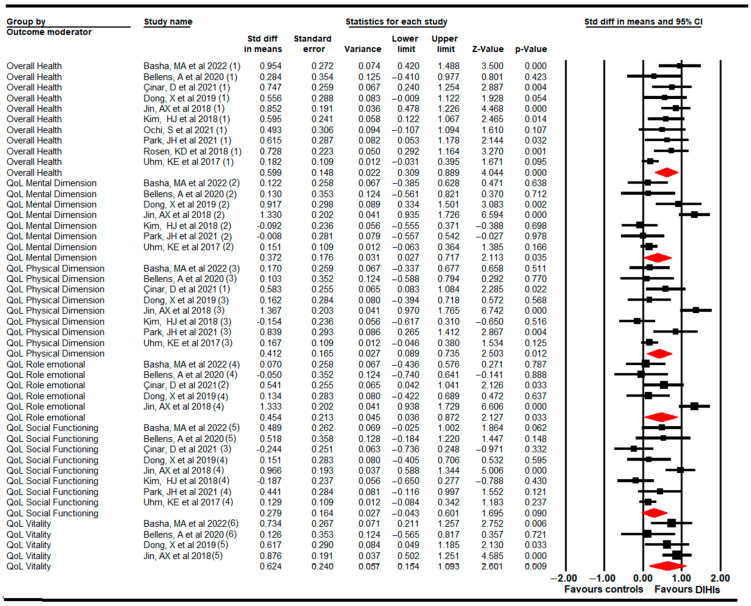
Forest plot of the effect of digital and interactive health interventions on different dimensions of quality of life [59,61,62,63,64,65,68,69,71,72].

We calculated the effect of DIHIs compared to classical training or care on different dimensions of quality of life (physical [59,61,63,65,68,69,71,72], mental [59,61,63,65,68,69,72], emotional [59,68,69,71,72], vitality [59,68,69,72], social functioning [59,61,63,65,68,69,71,72], and overall perceived health [59,61,62,63,64,65,68,69,71,72]. DIHIs showed a medium effect in increasing overall health (SMD, 0.6; 95% CI: 0.31, 0.89; *p* < 0.001), vitality (SMD, 0.62; 95% CI: 0.15, 1.1; *p* = 0.009), emotional dimensions (SMD, 0.45; 95% CI: 0.04, 0.87; *p* = 0.033), and physical dimensions (SMD, 0.41; 95% CI: 0.09, 0.74; *p* = 0.012), and a medium-low effect on mental dimensions (SMD, 0.37; 95% CI: 0.03, 0.72; *p* = 0.35). However, on the social functioning dimension, no statistically significant differences were found between DIHIs and controls (SMD, 0.28; 95% CI: −0.04, 0.6; *p* = 0.09) (Figure 6, Table 4). Heterogeneity was not present in any dimension, and a risk of publication bias was present in overall health perception, physical functioning, emotional functioning, social functioning, and vitality (details in Table 4 and Supplementary Figures S9–S14). The sensitivity analysis did not reveal substantial variations.

According to each dimension of quality of life, we obtained the following results in subgroup analyses: (1) VRBT (SMD, 0.76; 95% CI: 0.42, 1.11; *p* < 0.001) and SABT (SMD, 0.5; 95% CI: 0.3, 0.71; *p* < 0.001) were effective at increasing overall perceived health, although the effect of VRBT was larger; (2) for the physical dimension, only VRBT showed statistically significant differences in comparison with conventional training or care (SMD, 0.61; 95% CI: 0.04, 1.18; *p* = 0.038); (3) no statistically significant differences between VRBT or SABT were found for the mental or emotional dimensions; and (4) finally, VRBT produced more improvements in vitality than conventional training or care (SMD, 0.67; 95% CI: 0.27, 1; *p* = 0.001).

## 4. Discussion

Women diagnosed with BC often have a high level of disability associated with the progression of the disease, the adverse effect of chemical therapies, or motor disorders after BC surgery, such as lymphedema. In addition, the overall pain experienced is associated with anxiety and depression, decreasing the personal and social quality of life of patients. It is important to explore the effect of new therapies in comparison to other, classical approaches to the management of these symptoms. DIHIs, based on the use of virtual reality devices and smartphone education apps or games, show potential as an excellent therapy option for these patients. This research assessed the effect of these therapies on reducing UE motor disabilities, pain, anxiety, and depression and increasing quality of life in these patients. In addition, we wanted to investigate whether the effects of VRBT and SABT were similar, to determine what type of DIHI may be more effective for specific outcomes. We followed a detailed search strategy to obtain 20 studies that included two groups, in which one group received one physical exercise based on DIHIs, and the other received classical or conventional therapies.

Although previous reviews looked separately at the effects of VRBT and SABT in improving different symptoms of BC, our review is the first meta-analysis of the combined effects of these two virtual therapies that assesses the differences in effect between therapies. In comparison with other reviews, our study only included articles reporting on research with two groups. In addition, the number of studies (*n* = 20) and the number of participants (1613 patients with a mean age of 50.68 ± 3.7 years old) were both larger than in previous reviews, especially in terms of assessing the effect of VRBT, allowing an increased quality of evidence and potential for the generalization of our findings. Unlike previous reviews of SABT, our meta-analysis only included smartphone-app-based therapies with education apps or games that favored the practice of exercise in women with BC. Previous reviews differed from ours in that they combined studies of one group with studies of two groups and included not only applications that were based on the practice of exercise but also those designed for monitoring patient symptoms. Finally, one strength of our meta-analysis was that it performed a detailed and rigorous analysis of different variables related to the upper extremities and various dimensions of quality of life, unlike many previous reviews.

This meta-analysis presents the most complete assessment of the effect of DIHIs on the disabling of the upper extremities caused by lymphedema. Our findings suggest that DIHIs, especially non-immersive VRBT, are effective at increasing flexion, abduction, and external rotation shoulder range of motion. These results are in concordance with previous reviews, although Zhang H et al. only assessed abduction shoulder ROM [35], and the work of Bu X et al., which included four studies, included one study that only examined one group of patients [36]. In comparison, we included more studies that considered this outcome. In addition, VRBT reduced upper extremity disability in patients with lymphedema, increasing the functional capacity of the disabled arm. This result was free of heterogeneity and the risk of publication bias and contradicted a previous meta-analysis in which no significant differences were found between VRBT and classical therapy [34]. Another interesting result was that handgrip strength was improved using DIHI, as compared to conventional therapy, although the benefits were greater when physical exercise was followed and supervised using SABT. The impaired handgrip strength derived from BC-related lymphedema [75] constitutes one of the most prevalent long-term sequelae in surviving women [76] and can last for up to six years following surgery [77], decreasing the functional capacity and health status of these women [78]. From our findings, we postulate that, due to the improvement in handgrip strength, the practice of exercises supervised or monitored with smartphone apps or games is a good option for increasing the functional capacity of disabled upper extremities. In addition, and according to Tian Qi et al., VRBT did not have an effect on handgrip strength recovery. Finally, no virtual therapy produced a greater improvement than conventional therapy in terms of the circulatory symptoms of lymphedema or shoulder muscle strength. Furthermore, we found that conventional therapy based on strength training and proprioceptive neuromuscular facilitation may be superior to non-immersive VRBT.

Pain also was assessed in our review, showing that DIHIs can be very effective at reducing the pain level in women with BC by about three points on the pain visual analog scale. Among the two DIHIs assessed, VRBT showed a reduction of almost four points on the pain visual analog scale when compared to conventional therapies. This result is very interesting because VRBT was four times more effective than the MCID for VAS assessment (expected change of 10%) [79]. Our study, in addition to confirming the results of the meta-analysis by Bu X et al. [36], provided results with greater levels of evidence, accuracy, and generalization due to including a greater number of studies and participants. None of the subsequent reviews have discussed the effect of VRBT on pain. However, it is important to remark on the risk that publication bias present in this analysis underestimated our findings. Our review did not show statistically significant differences between smartphone-app-based therapy and conventional therapy for the reduction of pain. This may be because only two studies met the inclusion criteria for our review, which focused on the practice of physical exercise through SABT.

Anxiety and depression are two psychological symptoms that can disable women with BC. Along with pain, anxiety is suffered by approximately 50% of patients [22], and depression appears in 97% [35], so it is important to find therapies that reduce their impact. Our findings revealed that DIHIs were effective at reducing anxiety. In comparison with previous reviews, VRBT and SABT were both effective, although the reduction in anxiety was greater when VRBT was employed. Our findings were in agreement with previous reviews of virtual reality and smartphone devices [32,35]. With respect to depression, DIHIs produced a large reduction, which was greater without the risk of publication bias, as demonstrated by trim-and-fill estimation. Our subgroup analysis revealed that the practice of exercise using VRBT may have been better than SABT at reducing depression symptoms in comparison to conventional therapies. Our findings were in agreement with previous reviews [34,36], and although we included the same number of studies, our studies all included research on two groups of patients; thus, they may be more accurate.

Finally, our study was the first review to assess the effect of DIHIs on quality of life and its different dimensions. Our findings showed that DIHIs increased overall health perception, as well as the physical, mental, emotional, and vitality dimensions. However, no therapy was better than conventional therapy at increasing social functioning. Considering the two therapies, VRBT and SABT were both effective at increasing overall health perception, although the effect of VRBT was larger. Subgroup analysis revealed that VRBT had a major effect on the physical and vitality dimensions, which may be related to the continuous body movement that physical exercise requires. Findings on the effect of SABT agreed with previous reviews [37], and this was the first meta-analysis to assess the effect of VRBT on quality of life.

The improvements produced by virtual environments, especially VRBT, in comparison to conventional therapy, training, or usual care, are due to the distraction power and the active physical activity required to perform the virtual tasks [67]. Distraction is defined as anything that is preoccupying to the person who is paying attention and distracts their attention from pain and other problems [67]. VRBT or SABT require the active and conscious participation of the person, which favors the immersion of the person in a parallel world that feels real, allowing them to forget their disabling situation thanks to the continuous challenges of active movement that virtual environments require. Several studies have reported that distraction reduces the consciousness of pain by altering nociceptive responses [80]. Oncology therapy based on chemotherapy or surgery produces high levels of pain, anxiety, depression, and low self-esteem in women. The use of virtual environments, via virtual reality devices or mobile apps, has shown a reduction in perceived pain during chemotherapy and painful procedures. Distraction allows women to feel relaxed before stressful therapies and increases motivation while performing the physical exercises required in rehabilitation, especially in UE-related lymphedema motor recovery. DIHIs may be contemplative (passive) or participatory (active), and a recent study showed that both modalities are effective at reducing pain and anxiety and increasing emotional status in women with BC during chemotherapy [70,81]. A study suggested the distraction power of VRBT; a low level of pain was found when VRBT was used with opioids, with respect to no therapy or opioids alone [82]. DIHIs can actuate in different neuroanatomic areas related to pain perception (known as the “pain matrix”), such as the anterior cingulate cortex, the insula, the thalamus, and the primary and secondary somatosensory cortexes [82,83]. The distraction power of DIHIs affects the neurophysiological networks between the visual and somatosensory systems, diverting attention and leading to a slower response to incoming pain signals [25,84]. Interacting with DIHIs can favor the proliferation of positive emotions that produce endogenous pain reduction and a feeling of well-being.

The findings of this meta-analysis may have interesting applications in clinical practice. VRBT allows the practice of exercises in safe environments (homes and clinical centers) to help restore motor function in upper-extremity-related lymphedema. Practicing exercises in ludic and motivating virtual environments can increase adherence to therapy relative to classical rehabilitative therapies. VRBT can adapt the type of videogame (immersive or non-immersive), as well as the duration of the exposure and its difficulty, to the preferences of each patient, favoring the proliferation of positive feelings that allow the patient to achieve objectives without paying attention to their motor alterations or pain. On the other hand, SABT allows patients to practice supervised exercise anywhere, monitor the development of their practice, and obtain real-time feedback. These two therapeutic options are cheap, easily accessible for both patients and health professionals, and usually have few adverse effects.

Although our review presented important findings and has several strengths, some limitations must be considered. The first is that our meta-analysis included both quasi-experimental and randomized controlled trial studies, which could affect the generalization of our findings. However, the sensitivity analysis did not show variation in the effects according to study design. Secondly, the low number of studies included (i.e., those that met the inclusion criteria) may reduce the accuracy of our findings, although this review included more studies with experimental and comparison groups. Another limitation is the low number of comparisons in some meta-analyses, leading to very-low-quality evidence of those findings. In addition, the risk of performance and detection bias was large, and the risk of selection bias was moderate. The risk of publication bias present in some meta-analyses may change the reported effects of therapy, as shown by trim-and-fill estimation. Finally, all assessments were carried out over a short period of time, so no conclusions could be reached on effectiveness in the medium or long term. Future studies should evaluate the effects of physical exercise with DIHIs in the medium and long term, include a larger number of participants, and assess possible combined effects when paired with conventional therapy.

## 5. Conclusions

Our findings revealed that DIHIs, specifically VRBT and smartphone-app-based therapy, were effective at improving upper extremity motor disability related to lymphedema, pain, anxiety, depression, and quality of life in women with BC. Regarding lymphedema-associated disability, non-immersive VRBT increased flexion, abduction, external rotation range of motion, and upper extremity function. Practicing exercises using SABT was found to be more effective than conventional training for improving handgrip strength. Regarding pain, DIHIs, especially VRBT, were able to reduce pain levels by approximately four points. Regarding anxiety and depression, DIHIs using VRBT were more effective than conventional therapies. Finally, VRBT and SABT produced increases in overall health perception, with a stronger effect for VRBT. In addition, the physical, mental, emotional, and vitality dimensions of quality of life were improved using DIHIs. Our findings suggest that VRBT may be more recommendable than SABT for the management of BC-related disabling symptoms.

## Figures and Tables

**Table 1:** Search strategies used for each database.

Database	Search Strategy
PubMed Medline	(breast neoplasms[mh] or breast neoplasm*[tiab] or breast cancer*[tiab] or breast tumor*[tiab] or mammary cancer*[tiab] or breast cancer lymphedema[mh] or breast cancer lymphedema[tiab] or postmastectomy lymphedema[tiab]) AND (virtual reality[mh] OR virtual reality[tiab] OR virtual reality exposure therapy[mh] OR virtual reality exposure therapy[tiab] OR exergam*[tiab] or videogam*[tiab] or mobile applications[mh] or mobile application*[tiab] or mobile app*[tiab] or smartphone app*[tiab] or mobile game*[tiab] or smartphone game*[tiab])
SCOPUS	TITLE-ABS-KEY (“breast neoplasm” OR “breast cancer” OR “breast tumor” OR “mammary cancer” OR “breast cancer lymphedema” OR “postmastectomy lymphedema”) AND TITLE-ABS-KEY (“virtual reality” OR “virtual reality exposure therapy” OR “mobile applications” OR “mobile app” OR “mobile game” OR “videogame” OR “exergame”)
Web of Science	(*breast cancer*) (Topic) AND (*virtual reality* OR *mobile applications* OR *mobile app*) (Topic)
PEDro	(virtual reality) AND (breast cancer) (mobile) AND (breast cancer)
CINAHL Complete	AB (breast cancer or breast neoplasm or breast carcinoma or breast tumor) AND AB (virtual reality or smartphone applications or mobile apps)
SciELO	(breast cancer) AND (virtual reality OR smartphone)

**Table 2:** Main characteristics of the studies included and qualitative individual findings.

			Experimental Group		Control Group		Outcomes		
Study	K	Number of Patients	Sample Characteristics	Intervention Characteristics	Sample Characteristics	Intervention Characteristics	Outcomes	Test	Qualitative Findings
Aguirre-Carvajal, M. et al. 2015 [55] (Mexico) Design: Quasi-experimental (NB) Setting: Hospital Carlos Van Bürende Valparaíso (Chile) Funding: No	2	77 post-mastectomy female patients (58.76 ± 1.46 years old)	41 (57.66 ± 1.65 years old)	Nintendo Wii^®^ videogames for one month, 3 times per week and 32 min per session	36 (60.03 ± 2.51 years old)	Conventional care	Pain	VAS	Both groups improved, although the Wii group reported a greater reduction in pain
							UE function	Quick DASH-9 Scale	Both groups improved, although the Wii group reported greater improvement
Atef, D. et al. 2020 [56] (Egypt) Design: Quasi-experimental (NB) Setting: Physical Therapy Department at the National Cairo Institute Funding: No	2	30 female patients between 40 and 65 years old	15 (54.07 ± 8.28 years old) with post-mastectomy lymphedema	Nintendo Wii^®^ sports videogames for a duration of 30 min, 2 sessions per week for 4 weeks	15 (53.07 ± 7.24 years old) with post-mastectomy lymphedema	Conventional physical training using PNF for a duration of 30 min, 2 sessions per week for 4 weeks	UE function	Quick DASH-9 Scale	Statistically significant differences in the Nintendo Wii^®^ group (*p* = 0.001) and in the PNF group (*p* = 0.003). No significant differences between groups (*p* = 0.935)
							Excess limb volume	Milliliters	Each group presented a statistically significant reduction in lymphedema (Nintendo Wii^®^ *p* = 0.001 and PNF *p* = 0.004). However, no statistically significant differences were found between the groups (*p* = 0.9)
Bani Mohammad, E. et al. 2019 [67] (Jordan) Design: RCT (NB) Setting: King Hussein Cancer Center Funding: Yes. Deanship of Scientific Research at the University of Jordan	2	80 female patients (51.99 ± 10.34 years old) in chronic phase	40	Immersive VR (“Ocean Rift” or “Happy Place”) after 15 min to give morphine therapy (peak time effect)	40	Conventional care	Pain	VAS	Statistically significant differences in each group (*p* < 0.001 and *p* < 0.001) and between groups (*p* < 0.001)
							Anxiety	STAI	Statistically significant differences in each group (*p* < 0.001 and *p* < 0.001) and between groups (*p* < 0.001), favoring VR
Basha, M.A. et al. 2022 (Egypt) [68] Design: RCT (SB) Setting: National Cancer Institute and El-Sahel Teaching Hospital (Cairo) Funding: No	16	60 female patients (50.45 ± 2.29 years old) with a mean time since diagnosis of 5 years	30 (48.83 ± 7 years old) with post-mastectomy lymphedema	Xbox Kinect dance and sports videogames over 8 weeks, 5 days per week and once per day	30 (52.07 ± 748 years old) with post-mastectomy lymphedema	Physical training combining stretching with resistance exercises using dumbbells for 8 weeks	Pain	VAS	Statistically significant differences favoring Xbox (*p* = 0.0001)
							UE function	DASH	Statistically significant differences favoring Xbox (*p* = 0.0004)
							Quality of life	SF-36	Statistically significant differences favoring Xbox only in general health (*p* = 0.0005). None in vitality, physical, mental, social, or emotional aspects (*p* > 0.05)
							Handgrip strength	Dynamometer	Statistically significant differences favoring the physical training group (*p* = 0.0002)
							Shoulder ROM	Degree	Greater improvements in flexion (*p* = 0.0001), abduction (*p* = 0.0001), and external rotation (*p* = 0.0001), favoring Xbox
							Shoulder strength	Kg	Greater improvements in flexion (*p* = 0.002), abduction (*p* = 0.0007), and external rotation (*p* = 0.004), favoring the physical training group
							Excess limb volume	Ml	Each group showed a statistically significant reduction in lymphedema (*p* < 0.0001 for both). However, no statistically significant differences were found between groups (*p* = 0.15)
Bellens, A. et al. 2020 (Turkey) [69] Design: RCT Pilot (NB) Setting: Multidisciplinary Breast Clinic of the Antwerp University Hospital Funding: Yes. MyCognition (one author)	9	46 female patients (51.8 ± 0.42 years old) with a mean time since diagnosis of 3.8 years	23 (51.5 ± 8 years old)	AquaSnap cognitive training videogame for a duration of 3 months of at least 3 times per week in addition to usual care	23 (52.1 ± 9.1 years old)	Usual care for 3 months	Anxiety/Depression	HADS	Greater reduction in anxiety and depression in the VR group (1.7 and 1.4 points, respectively)
							Pain	MOS-SF36	No statistically significant reduction in the control group (*p* > 0.05)
							Quality of life	MOS-SF36	Greater increase in physical functioning (7 points) and mental health (9.6 points) in the VR group than in the control group
Chirico, A. et al. 2019 (Italy) [70] Design: Quasi-experimental (NB) Setting: Fondazine G. Pascale (Naples, Italy) Funding: Yes. Sbarro Health Research Organization	4	92 female patients (55.69 ± 0.5 years old) in chronic phase	28 (55.18 ± 5.7 years old)	Walk, climb a mountain, swim in the sea, among others, in relaxed environments created on the Second Life^®^ platform using Immersive VR	30 (55.7 ± 5.26 years old)	Music therapy for 20 min, 5 min after the start of chemotherapy	Anxiety	SAI	Statistically significant differences between pre- and post-assessments in the VR group (*p* < 0.001) and the music therapy group (*p* < 0.001), but not in the control group (*p* = 0.179). Between groups, statistically significant differences were found between the VR and control groups (*p* < 0.001) and between the music therapy and control groups (*p* = 0.049). No statistically significant differences were found between the VR and music therapy groups (*p* > 0.05)
					34 (56.2 ± 6.79 years old)	Usual care			
							Depression	SV-POMS	Statistically significant differences between the VR and control groups (*p* < 0.001)
Çınar, D. et al. 2021 (Turkey) [71] Design: RCT (SB) Setting: State hospital in Turkey Funding: Yes. Mobile app by Balikesir Tuberculosis and Cancer Fight Asociation	5	64 female patients (45.7 ± 9 years old) with a mean time since diagnosis of 2.7 years	31 (45.9 ± 8.3 years old)	Mobile phone app-based training support (educational and relaxation exercises) in addition to usual care for 12 weeks	33 (45.5 ± 9.8 years old)	Usual care for 12 weeks	Anxiety	NCCNDTS	Statistically significant differences after therapy in the mobile app group (*p* = 0.004), but not in the control group (*p* = 0.082). Statistically significant differences between groups, favoring the mobile app group (*p* = 0.027)
							Quality of life	FACT-ES QLS	Statistically significant differences in total (*p* < 0.001), physical *p* < 0.0001), and emotional QoL (*p* = 0.0015) were shown in the mobile app group. Between groups, statistically significant differences, favoring the mobile group, were shown in total, physical, and emotional QoL
Dong, X. et al. 2019 (China) [72] Design: RCT (SB) Setting: The Second Hospital of Shandong University in China Funding: Yes. Specialized Key Subjects of China National S&T Fundamental Work	7	50 female patients (49.81 ± 2.55 years old) in chronic phase	26 (48 ± 5.54 years old)	Mobile phone app-based exercise and video exercises for 12 weeks, 3 times per week and 30 min per session	24 (51.63 ± 7.49 years old)	Usual care for 12 weeks	Quality of life	SF-36	In the exercise app group, statistically significant differences were found in global health (*p* = 0.024), vitality (*p* = 0.014), and mental health (*p* = 0.014). Between groups, statistically significant differences were found favoring the exercise app group in vitality (*p* = 0.009) and mental health (*p* = 0.001)
							Pain	SF-36	
Feyzioğlu, Ö. et al. 2020 (Turkey) [73] Design: RCT (SB) Setting: Okmeydanı Training and Research Hospital (Istanbul) Funding: No	9	36 female patients (50.92 ± 0.11 years old) with a chronic duration	19 (50.84 ± 8.53 years old)	Xbox 360 Kinect dance, sports, and fighting videogames for a duration of 35 min for 8 weeks. CT program added	17 (51 ± 7.06 years old)	Conventional physical therapy (usual care) for 8 weeks	Pain	VAS	Statistically significant improvement in both groups in pre–post assessment (*p* = 0.001 in each group). No differences between groups (*p* = 0.065)
							UEfunction	DASH Scale	Statistically significant improvement in both groups in pre–post assessment (*p* = 0.001 in each group). Between groups, statistically significant differences were found favoring the Xbox group (*p* = 0.025)
							Handgrip strength	Dynamometer	Statistically significant improvement in both groups in pre–post assessment (*p* = 0.001 in each group). No differences between groups (*p* = 0.302)
							Shoulder ROM	Degree	Greater improvements in flexion, abduction, and external rotation in both groups, but no statistically significant differences between them in flexion (*p* = 0.688), abduction (*p* = 0.793), or external rotation (*p* = 0.573)
							Shoulder strength	Kg	Greater improvements in flexion, abduction, and external rotation in both groups, but no statistically significant differences between them in flexion (*p* = 0.203), abduction (*p* = 0.532), or external rotation (*p* = 0.666)
Ghanbari, E. et al. 2021 (Iran) [74] Design: RCT (NB) Setting: Shiraz University of Medical Sciences (Shiraz) Funding: Yes. Shiraz University of Medical Sciences	2	77 female patients (46.45 ± 0.63 years old) in chronic phase	38 (46.9 ± 9.83 years old) in chronic phase	Mobile phone app-based training support (educational exercises) in addition to usual care for 4 weeks	39 (46 ± 8.8 years old) in chronic phase	Usual care for 4 weeks	Anxiety	STAI	Statistically significant reduction in the mobile app group (*p* < 0.001) and increase in anxiety in the control group (*p* = 0.34)
Handa, S. et al. 2020 (Japan) [57] Design: RCT (NB) Setting: Showa University Hospital Breast Cancer (Japan) Funding: No	2	95 (49.9 ± 9.7 years old)	47 (49.9 ± 10.2 years old) in chemotherapy phase	Mobile app support training for 3 weeks (4 courses of chemotherapy)	48 (49.9 ± 9.2 years old) in chemotherapy phase	Usual care	Anxiety	HADS-A	In both groups, the level of anxiety increased. However, the level of anxiety was lower in the usual care group, with statistically significant differences between groups (*p* = 0.08)
							Depression	HADS-D	Usual care group did not show a statistically significantly reduced level of depression (*p* > 0.05), with no differences between groups (*p* = 0.35). The mobile app group showed increased depression (*p* > 0.05)
Jimenez, Y.A. et al. 2018 (Australia) [58] Design: Quasi-experimental (NB) Setting: Crown Princess Mary Cancer Centre, Westmead Hospital, Australia Funding: University of Sydney’s postgraduate research support	1	37 female patients between 35 and 74 years old (major part between 45 and 54 years old)	19	VR education using VERT system. A total of 18 sessions of 1 h were carried out	18	Usual care education	Anxiety	STAI	VR further reduced the level of anxiety, but no statistically significant differences were found between groups (*p* = 0.217)
Jin, A.X. et al. 2018 [59] (China) Design: RCT (NB) Setting: Zhejiang Provincial People’s Hospital (China) Funding: No	6	120 female patients in chronic phase	60	VR exercises using the Omaha system for 3 months	60	Conventional physical training for 3 months	QoL	SF-36	In the VR group, there was an increase in total, physical, mental, emotional, vitality, and social QoL. Statistically significant differences in these dimensions appeared, favoring the VR group (*p* < 0.05)
Jin, A. et al. 2018 (China) [60] Design: RCT (SB) Setting: Zhejiang Provincial People’s Hospital (China) Funding: No	3	72 female patients in chronic phase	38	VR-based training for 3 months, twice per day, 15–30 min per session	38	Conventional physical training for 3 months	Shoulder ROM	Degree	Patients who performed VR rehabilitation showed greater increases in shoulder flexion, abduction, and external rotation as compared to the conventional physical training group (*p* < 0.05)
Kim, H.J. et al. 2018 (Korea) [61] Design: RCT (NB) Setting: Chung-Ang University Hospital (Korea) Funding: Yes. Nexon 2014 and Korea Creative Content Agency	3	77 female patients (50.95 ± 1.6 years old) with a mean duration of disease of 13.35 years	34 (49.8 years old) with a mean duration of disease of 13.5 years	Mobile game for a duration of 3 weeks, 3 days per week and more than 30 min per session.	38 (52.1 years old) with a mean duration of disease of 13.2 years	Conventional therapy for 3 weeks	Anxiety	STAi	Low level of anxiety in the mobile app group (*p* = 0.11) and no statistically significant differences between groups (*p* = 0.21)
							Depression	BDI	Depression increases in both groups (*p* > 0.5) and no statistically significant differences between groups (*p* = 0.99)
							Quality of life	WHO QoL-BREF Scale	The mobile app group showed higher QoL than the conventional therapy group (*p* = 0.01). Between groups, greater improvements were found favoring conventional therapy in physical (*p* = 0.03), mental (*p* = 0.2), and social QoL (*p* = 0.67)
Ochi, E. et al. 2021 (Japan) [62] Design: RCT (SB) Setting: National Cancer Center Hospital (Tokyo) Funding: Yes. National Cancer Centre Research and Development Fund	2	44 female patients (48.5 years old) in chronic phase	21 (48 ± 6 years old) with more than 19 months of evolution	Smartphone app exercise training guidance for 12 weeks and 3 times per week	23 (49 ± 5 years old)	Usual care for 13 weeks	Handgrip strength	Dynamometer	More improvements in the app group, but with no statistically significant differences between groups (*p* = 0.53)
							Quality of life	QoL (EQ-5D)	No statistically significant differences between groups (*p* = 0.25), although app groups improved more
Park, J.H. et al. 2022 (South Korea) [63] Design: Quasi-experimental (NB) Setting: Breast Cancer Center, Ajou University Medical Center, Suwon (South Korea) Funding: Yes. Basic Science Research Program through the National Research Foundation of Korea	4	51 (42.78 ± 4.7 years old) in chronic phase	27 (42.78 ± 4.7 years old)	Smartphone app education for 12 weeks	24 (45 ± 5 years old)	Conventional education for 12 weeks	Quality of life	FACT-G	Statistically significant differences favoring the app group in social support (*p* = 0.04) and mental QoL (*p* = 0.003). The app group improved in all QoL dimensions in the pre–post assessment
Rosen, K.D. et al. 2018 [64] (United States) Design: RCT (NB) Setting: University of Texas, San Antonio Funding: Yes. ThriveWell Cancer Foundation and Graduate Student Research Award from the University of Texas at San Antonio	1	87 female patients (52.31 ± 1.28 years old), duration of disease between 3 and 5 years	39 (51.4 ± 10.73 years old)	Mobile app mindfulness (Headspace) mediation training for 8 weeks	48 (53.22 ± 9.91 years old)	Usual care	Quality of life	FACT-B	After intervention, QoL was higher in the app group than in the control, with statistically significant differences favoring the app group (*p* < 0.01)
Uhm, K.E. et al. 2017 [65] (Korea) Design: Quasi-experimental (NB) Setting: Universities and hospitals in Korea Funding: Yes. National Information Society Agency (Korea)	6	339 female patients (50.3 ± 9.5 years old) with more than 2 years of evolution	167 (49.3 ± 8 years old)	Mobile app exercise training (mHealth), including aerobic and resistance exercise for 12 weeks	172 (51.3 ± 10.7 years old)	Conventional exercises for 12 weeks	Handgrip strength	Dynamometer	Statistically significant improvements in the app (*p* < 0.05) and control (*p* < 0.05) groups. No statistically significant differences between groups (*p* > 0.5)
							Pain	VAS	Statistically significant reduction in app (*p* < 0.05) and control (*p* < 0.05) groups. No statistically significant differences between groups (*p* > 0.22)
							QoL	EORTC QLQ-C30	Global QoL statistically improved in the app (*p* < 0.05) and control (*p* < 0.05) groups. Between groups, no statistically significant differences were found in global (*p* = 0.746), physical (*p* = 0.337), emotional (*p* = 0.42), or social (*p* = 0.608) QoL
Zhu, L. et al. 2019 [66] (China) Design: RCT Setting: Hospital Funding: No	3	80 female patients in chronic phase	40	VR exercises for shoulder and hand rehabilitation. Three months, twice per day, 15–30 min each day	40	Conventional exercises for 3 months	Shoulder ROM	Degree	The VR rehabilitation group showed greater increases in shoulder flexion, abduction, and external rotation as compared to the conventional physical training group (*p* < 0.05)

Abbreviations: K, number of comparisons; RCT, randomized controlled trial; NB, not blinded; SB, single-blinded; PNF, proprioceptive neuromuscular facilitation; UE, upper extremity; VR, virtual reality; VAS, Visual Analog Scale; STAI, State-Trait Anxiety Inventory; SAI, State Anxiety Inventory; NCCNDTS, NCCN Distress Thermometer Scale; SV-POMS, Profile of Mood State-short version; HADS-A/D, Hospital Anxiety and Depression Scale (anxiety and depression subscales); BDI, Beck Depression Inventory; FACT-ES QLS, FACT-ES quality of life scale; FACT-B, Functional Assessment of Cancer Therapy—Breast version 4; EORTC QLQ-C30, European Organization for Research and Treatment of Cancer Quality of Life Questionnaire Core 30; WHO QoL-BREF Scale, World Health Organization Quality of Life-BREF Scale 3.3. Risk of bias in the studies included.

**Table 3:** Risk of bias assessment of the included studies.

STUDY	Selection Bias		Performance Bias	Detection Bias	Attrition Bias	Reporting Bias	Other Bias
	Random Sequence Generation	Concealment Randomization Sequence	Blinding of Participants	Blinding of Assessors	Incomplete Outcome Data	Selective Reporting	Other, Ideally Prespecified
**Aguirre-Carvajal, M. et al. 2015 [55]**	+	+	+	+	−	?	?
**Atef, D. et al. 2020 [56]**	+	+	+	+	−	−	?
**Bani Mohammad, E. et al. 2020 [67]**	−	−	+	+	−	−	−
**Basha, M.A. et al. 2022 [68]**	−	−	+	−	−	−	−
**Bellens, A. et al. 2020 [69]**	−	−	+	+	?	−	−
**Chirico, A. et al. 2019 [70]**	+	+	+	+	−	−	?
**Çinar, D. et al. 2021 [71]**	−	?	+	−	−	−	−
**Dong, X. et al. 2019 [72]**	−	−	+	−	−	−	−
**Feyzioğlu et al. 2020 [73]**	−	−	+	+	−	?	−
**Ghanbari, E. et al. 2021 [74]**	−	−	+	+	−	−	−
**Handa, S. et al. 2020 [57]**	−	?	+	+	−	−	−
**Jimenez, Y.A. et al. 2018 [58]**	+	+	+	+	−	−	?
**Jin, A.X. et al. 2018 [59]**	−	−	+	+	−	−	−
**Jin, A. et al. 2018 [60]**	−	−	+	+	−	−	−
**Kim, H.J. et al. 2018 [61]**	−	−	+	+	−	−	−
**Ochi, E. et al. 2021 [62]**	−	−	+	−	−	−	−
**Park, J.H. et al. 2022 [63]**	+	+	+	+	−	−	?
**Rosen, K.D. et al. 2018 [64]**	−	−	+	+	−	−	−
**Uhm, K.E. et al. 2017 [65]**	+	+	+	+	−	−	?
**Zhu, L. et al. 2019 [66]**	−	−	+	+	−	−	−

Abbreviations: “+” = high risk of bias, “−” = low risk of bias, “?” = uncertain risk of bias.

**Table 4:** Main findings.

		Findings Summary												
		Effect Size						Heter			Publication Bias			
		K	N	N_s_	SMD	95% CI	*p*	Q (df)	I^2^ *(p)*	Risk	Funnel Plot (Egger *p*)	Trim and Fill		Risk
												Adj SMD	% var	
UPPER EXTREMITY	Flexion ROM	4	248	62	1.92	1.16 to 2.68	<0.001	3.18 (3)	5.72% (0.36)	No	Symmetric (0.92)	1.92	0%	No
	Abduction ROM	4	248	62	1.66	0.91 to 2.42	<0.001	3.78 (3)	20.71% (0.29)	Low	Asymmetric (0.48)	1.35	19%	High
	External Rotation ROM	4	248	62	1.1	0.36 to 1.85	0.004	3.17 (3)	5.64% (0.35)	No	Symmetric (0.97)	1.1	0%	No
	Flexion Strength	2	86	43	−0.03	−1.66 to 1.62	0.97	1 (1)	0% (0.32)	No	NP	NP	NP	Possible
	Abduction Strength	2	86	43	−0.2	−1.83 to 1.44	0.81	1 (1)	0% (0.32)	No	NP	NP	NP	Possible
	External Rotation Strength	2	86	43	0.1	−1.54 to 1.74	0.9	1 (1)	0% (0.32)	No	NP	NP	NP	Possible
	Excessive Limb Volume	2	90	45	−0.18	−0.66 to 0.3	0.46	1 (1)	0% (0.32)	No	NP	NP	NP	Possible
	Handgrip Strength	4	477	119	0.4	0.21 to 0.59	<0.001	50.6(3)	67% (0.0001)	Large	Asymmetric (0.15)	0.67	65%	High
	Function	4	203	51	−0.72	−1.31 to −0.13	0.017	3.17 (3)	5.5% (0.35)	No	Symmetric (0.97)	−0.72	0%	No
PAIN		8	758	95	−0.8	−1.31 to −0.26	0.003	7.53 (7)	7.14% (0.38)	No	Asymmetric (0.08)	−0.9	13%	High
ANXIETY		9	660	73	−1.02	−1.71 to −0.34	0.003	11.8 (8)	32% (0.16)	Medium	Asymmetric (0.02)	−1.35	32%	High
DEPRESSION		5	402	80	−1.57	−3.1 to −0.08	0.039	8.96(4)	46% (0.06)	Medium	Asymmetric (0.01)	−2.05	29%	High
QUALITY OF LIFE	Overall Health Perception	10	888	89	0.6	0.31 to 0.89	<0.001	5.8 (9)	0% (0.76)	No	Asymmetric (0.02)	0.35	42%	High
	Physical Role	8	759	95	0.41	0.08 to 0.74	0.012	6.4 (7)	0% (0.5)	No	Asymmetric (0.3)	0.5	19%	High
	Mental Role	7	695	99	0.37	0.03 to 0.72	0.035	5.76 (6)	0% (0.45)	No	Symmetric (0.73)	0.37	0%	No
	Emotional Role	5	292	58	0.45	0.04 to 0.87	0.033	3.24 (4)	0% (0.52)	No	Asymmetric (0.01)	0.53	18%	High
	Social Functioning	8	759	95	0.28	−0.04 to 0.6	0.09	6.62 (7)	0% (0.47)	No	Asymmetric (0.34)	0.16	47%	High
	Vitality	4	228	57	0.62	0.15 to 1.1	0.009	3.06 (3)	1.8% (0.38)	No	Asymmetric (0.03)	0.82	32%	High

Abbreviations: K, number of comparisons; N, number of participants in each meta-analysis; Ns, number of participants per study; SMD, standardized mean difference; 95% CI, 95% confidence interval; *p*, *p*-value; Q, Q-test; df, degree of freedom; I2, degree of inconsistency; Adj, adjusted; ROM, range of motion; NP, not possible to calculate.

## Data Availability

Data are available requesting to corresponding author.

## Supplementary Materials

The following supporting information can be downloaded at: https://www.mdpi.com/article/10.3390/cancers14174133/s1, Figure S1: Funnel plot for shoulder flexion movement; Figure S2: Funnel plot for shoulder abduction movement; Figure S3: Funnel plot for shoulder external rotation movement; Figure S4: Funnel plot for handgrip strength; Figure S5: Funnel plot for upper extremity function; Figure S6: Funnel plot for pain; Figure S7: Funnel plot for anxiety; Figure S8: Funnel plot for depression; Figure S9: Funnel plot for overall health perception; Figure S10: Funnel plot for physical quality of life; Figure S11: Funnel plot for mental quality of life; Figure S12: Funnel plot for emotional quality of life; Figure S13: Funnel plot for social functioning; Figure S14: Funnel plot for vitality.
