# Supplementary material for: Digital and Interactive Health Interventions Minimize the Physical and Psychological Impact of Breast Cancer, Increasing Women’s Quality of Life: A Systematic Review and Meta-Analysis
Source: Cancers (Basel). 2022 Aug 26;14(17):4133. doi: 10.3390/cancers14174133 (PMC9454975; doi:10.3390/cancers14174133)
Supplement: Supplementary file 1 [file cancers-14-04133-s001.zip › cancers-1823109-supplementary.pdf]

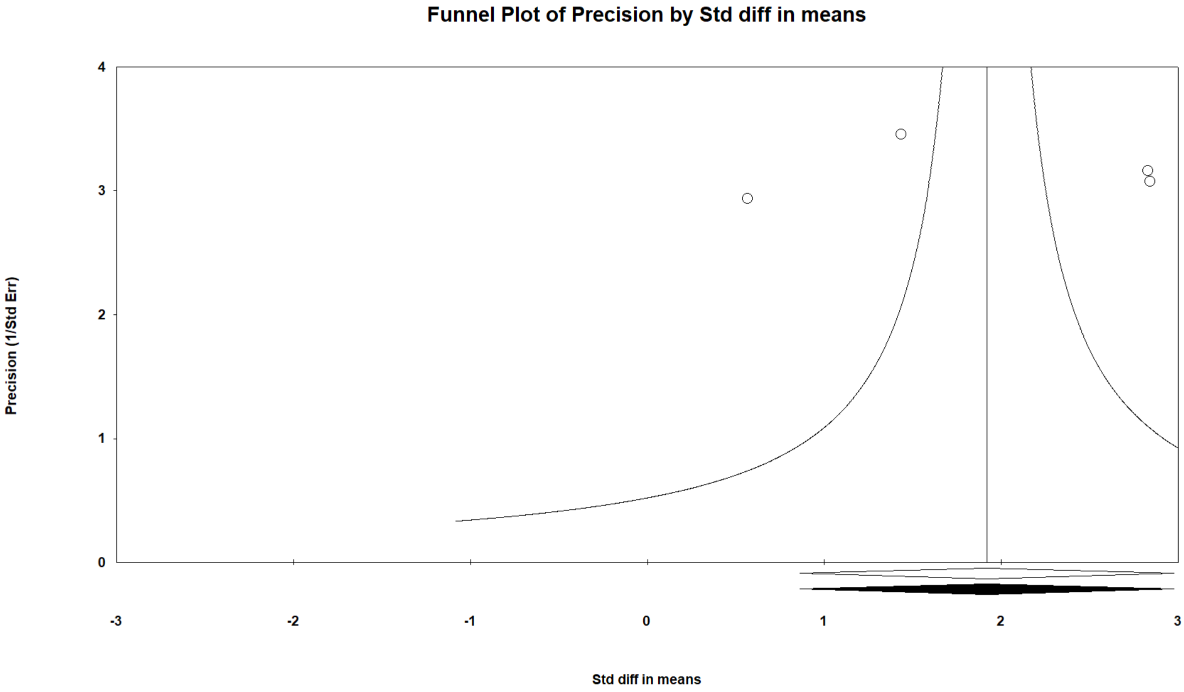

Figure S1. Funnel plot shoulder flexion movement.

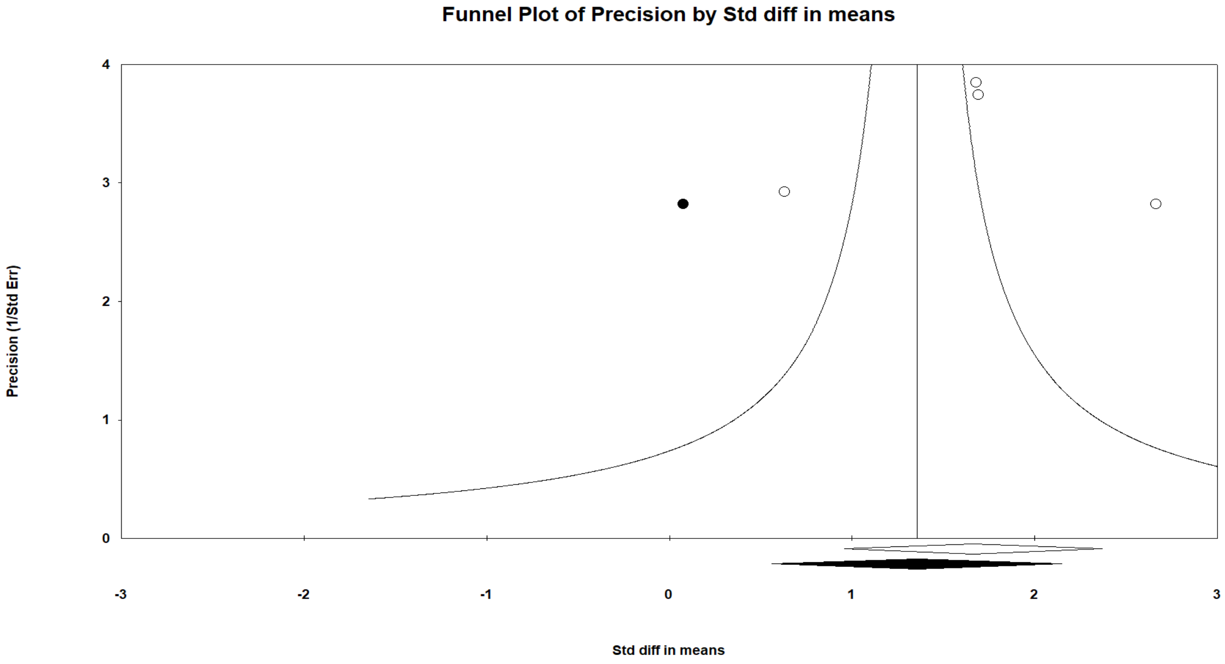

Figure S2. Funnel plot shoulder abduction movement

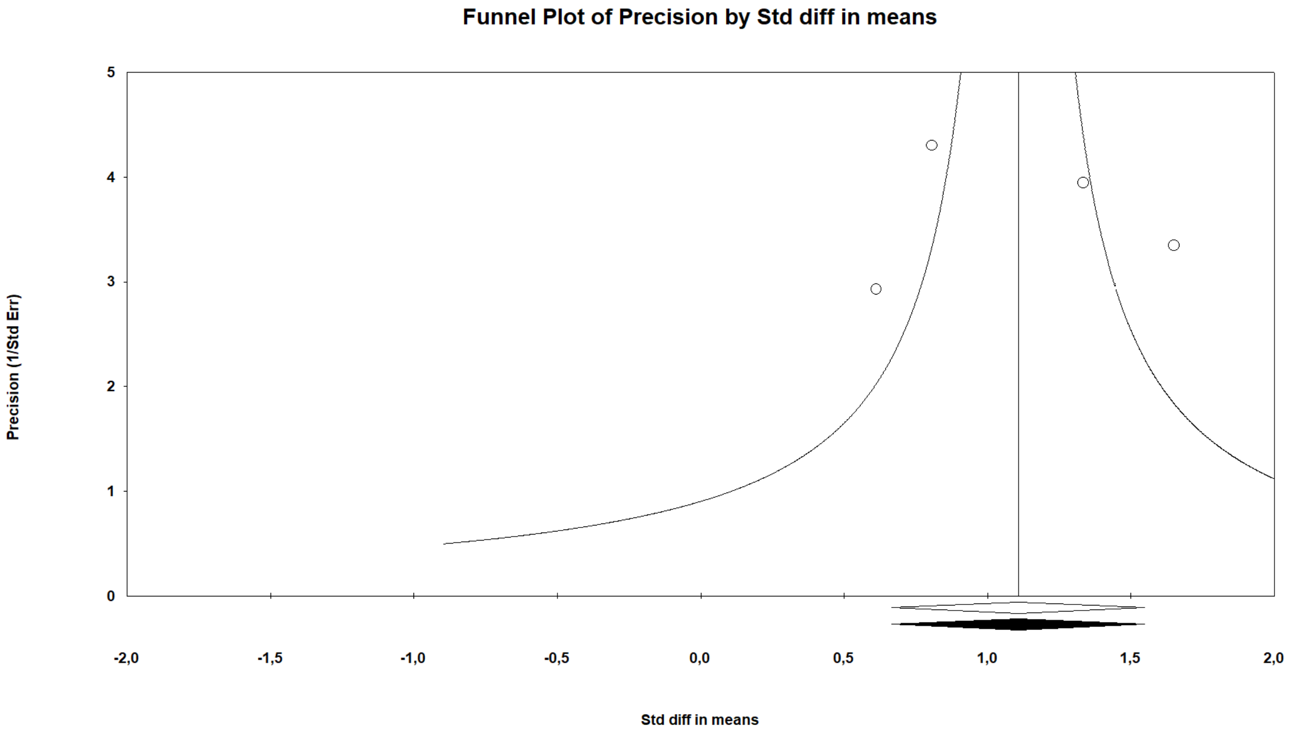

Figure S3. Funnel plot shoulder external rotation movement

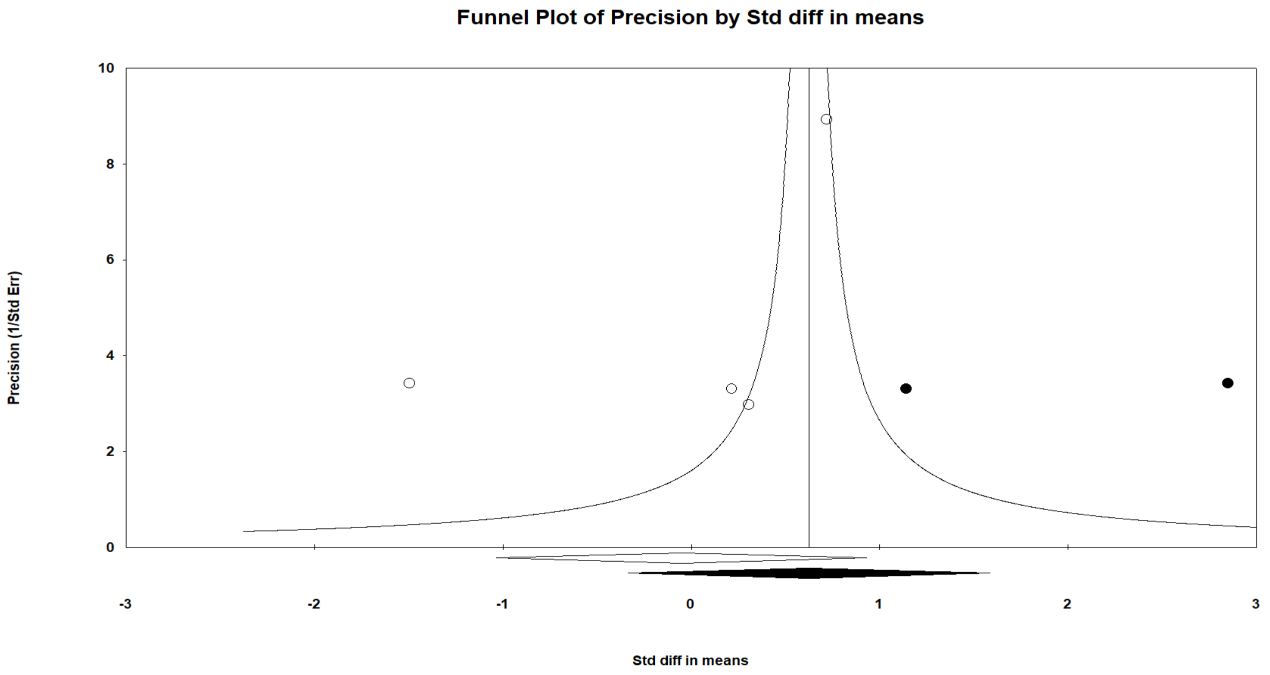

Figure S4. Funnel plot handgrip strength

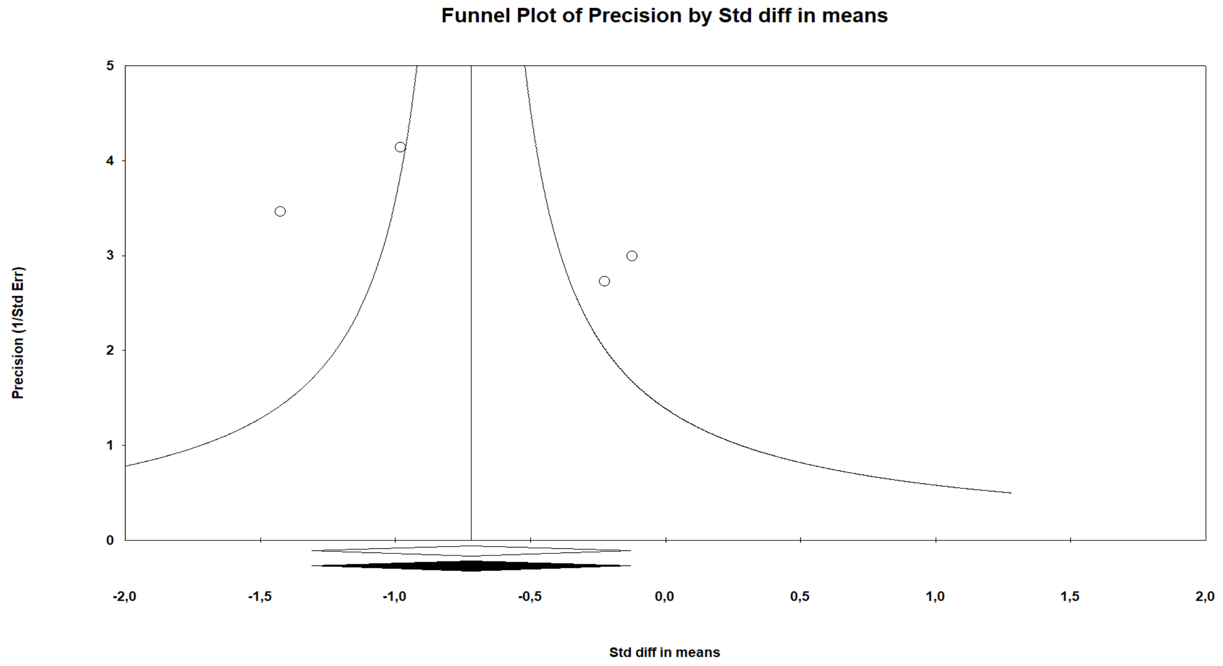

Figure S5. Funnel plot upper extremity function

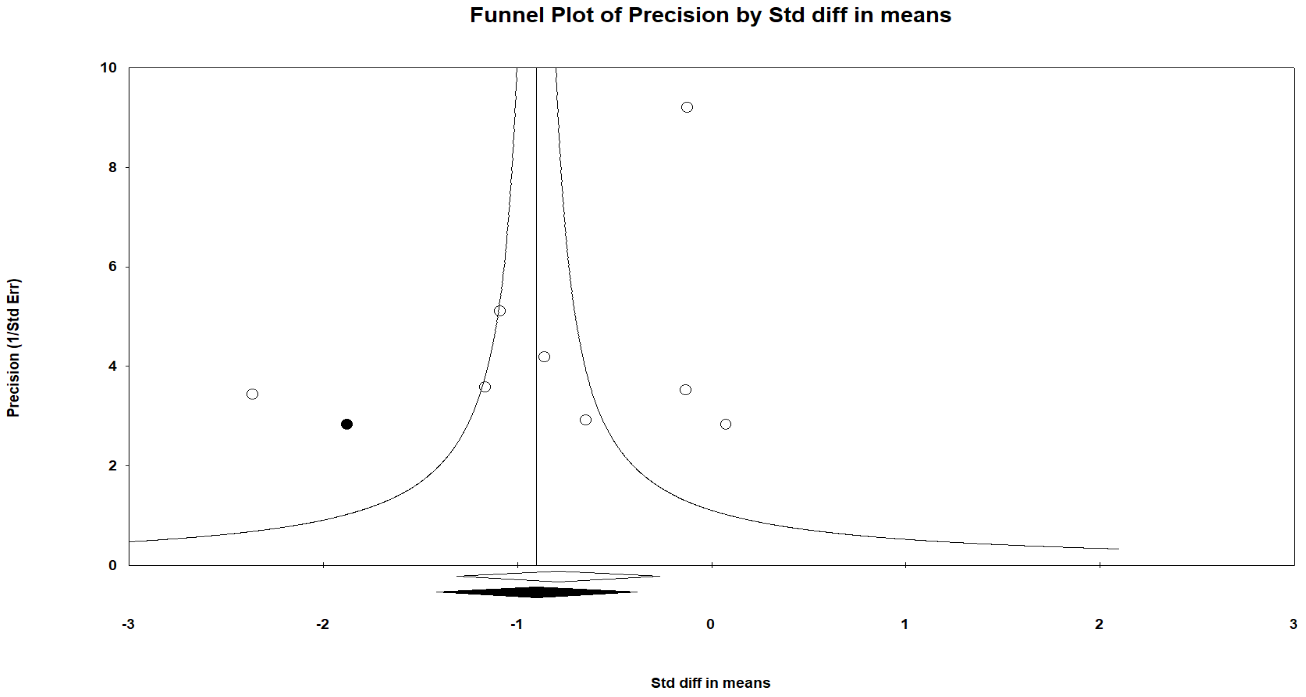

Figure S6. Funnel plot pain

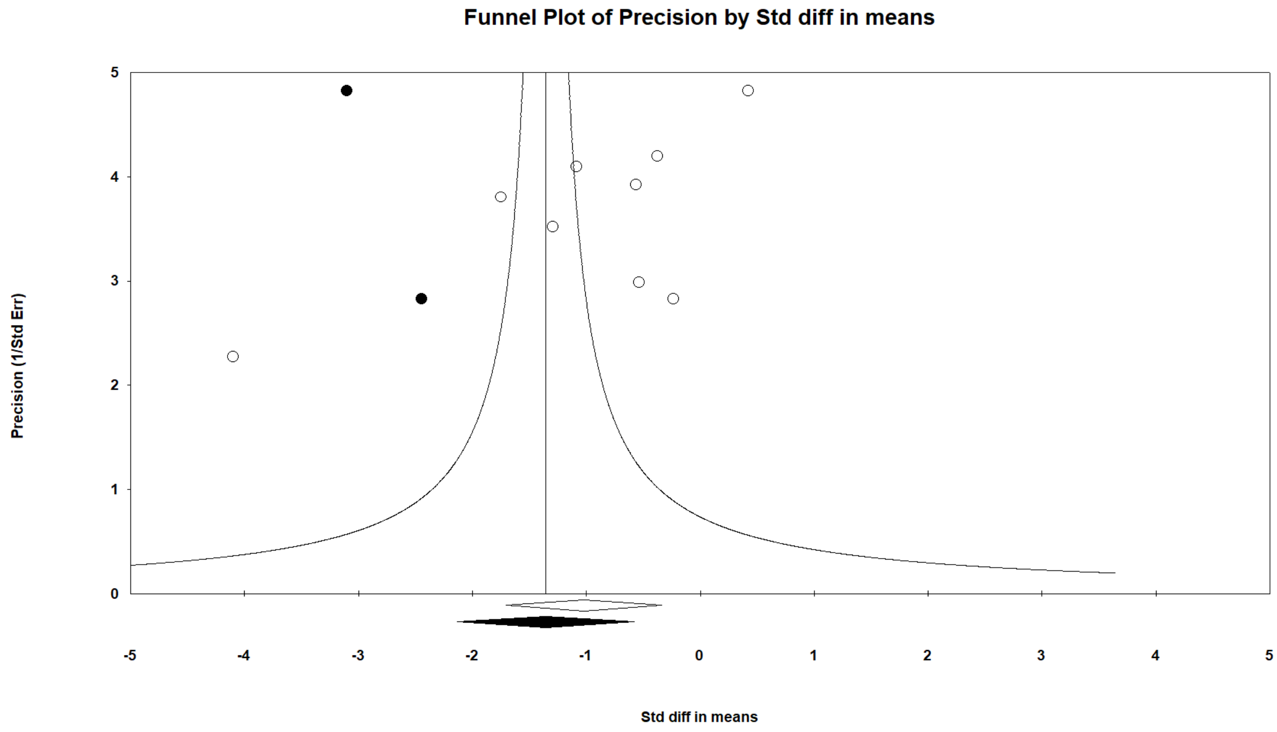

Figure S7. Funnel plot anxiety

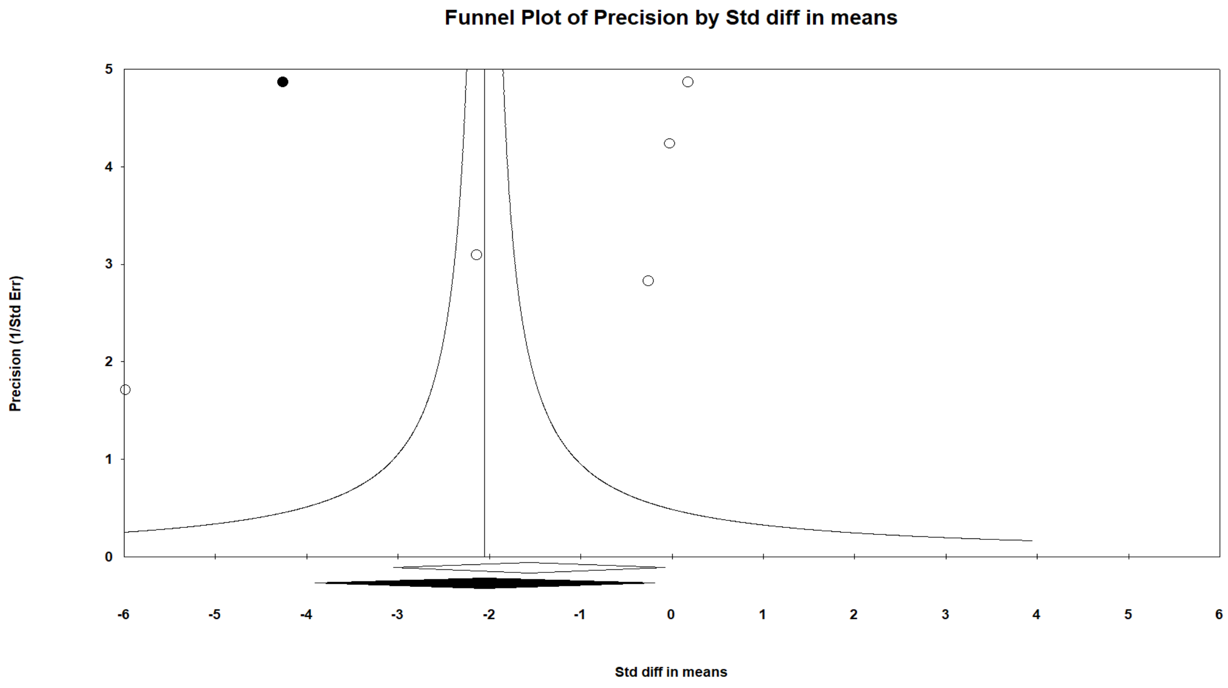

Figure S8. Funnel plot depression

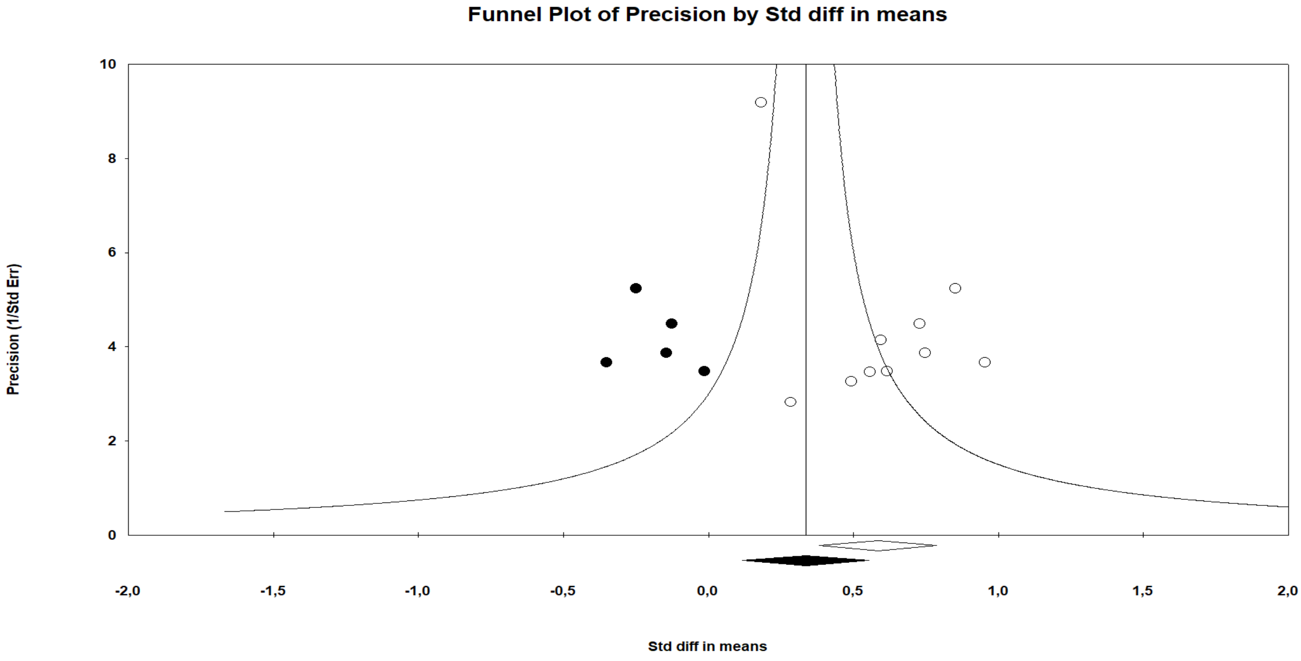

Figure S9. Funnel plot overall health's perception

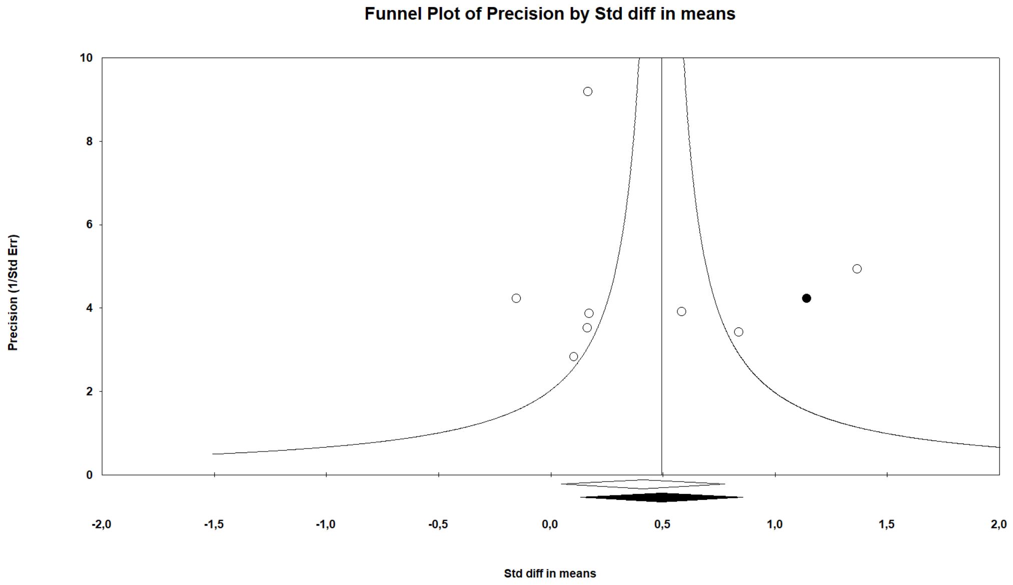

Figure S10. Funnel plot physical quality of life

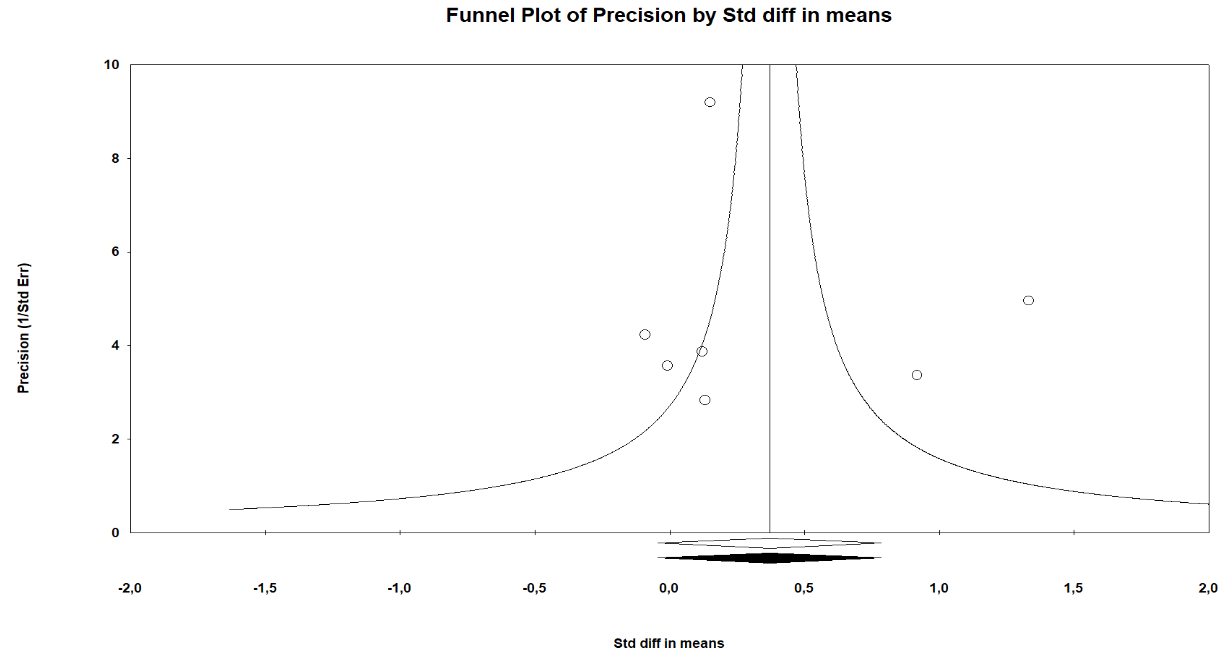

Figure S11. Funnel plot mental quality of life

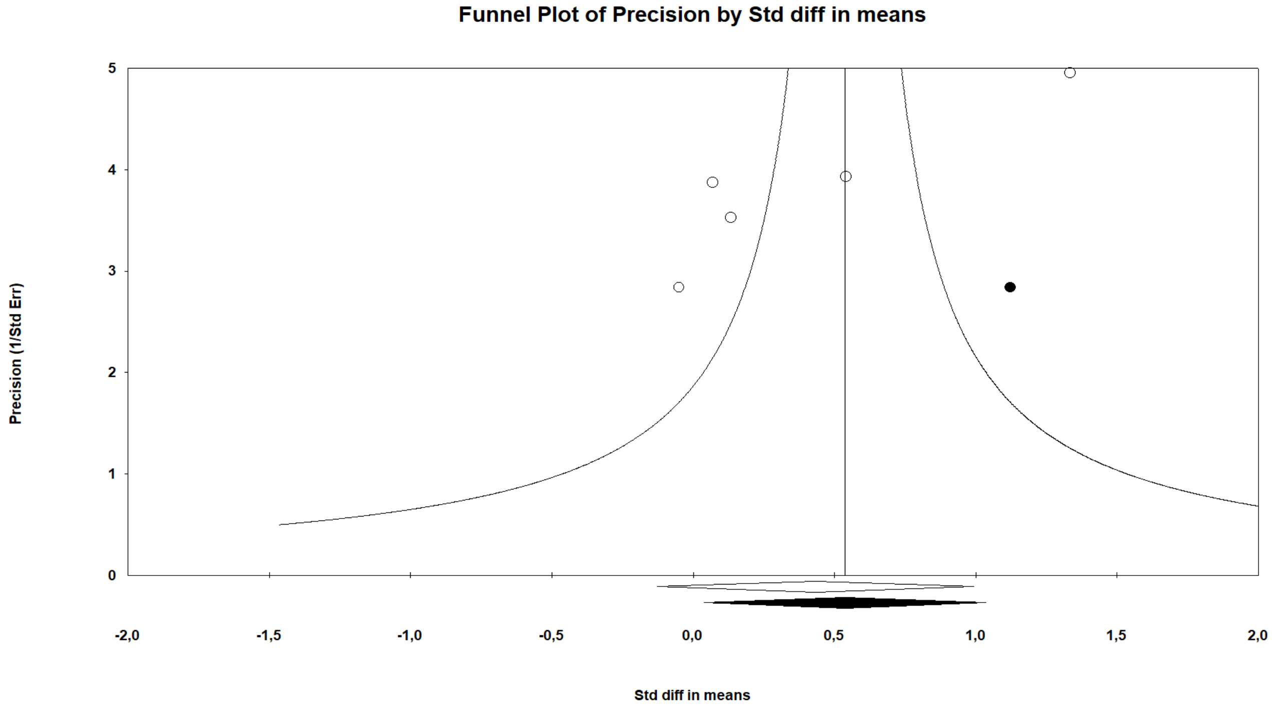

Figure S12. Funnel plot emotional role quality of life

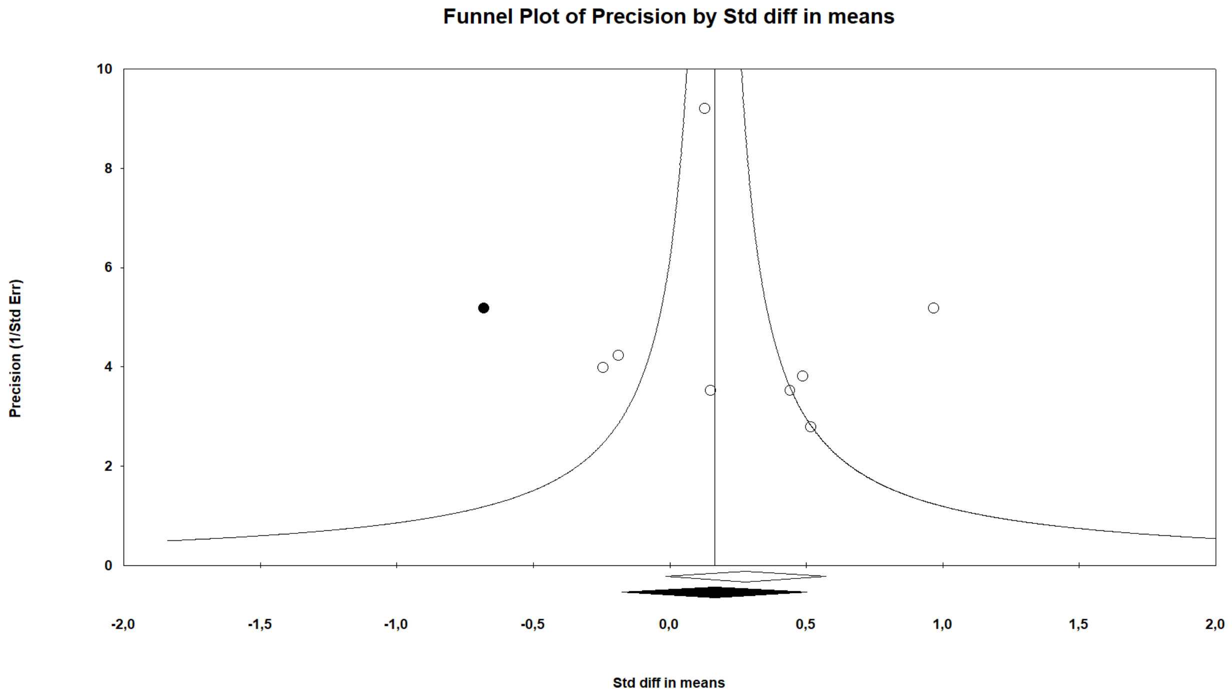

Figure S13. Funnel plot social functioning

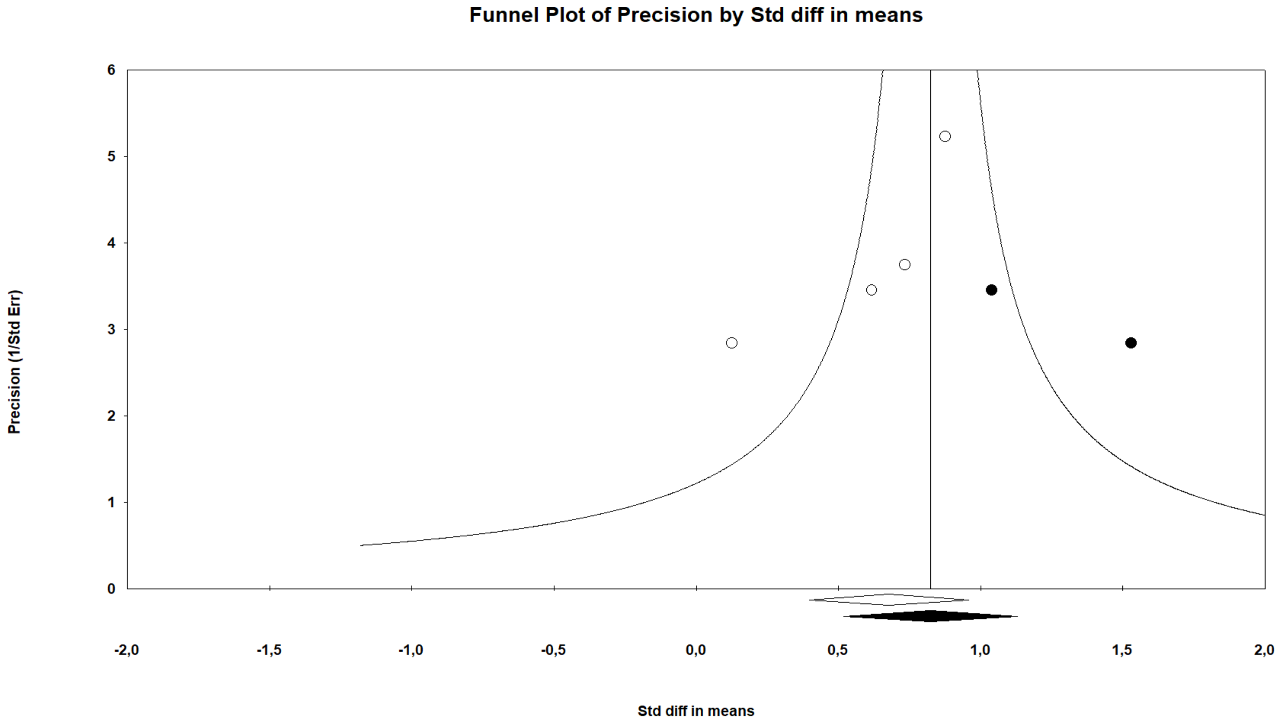

Figure S14. Funnel plot vitality
